# Frequency-specific microstate correlates of ciprofol-induced alterations of consciousness

**DOI:** 10.3389/fnins.2026.1781321

**Published:** 2026-04-07

**Authors:** Fei Yan, Yansong Li, Ni Xiong, Yun Zhang, Yaomin Zhu

**Affiliations:** 1 Department of Anesthesiology and Surgery, The First Affiliated Hospital of Xi’an Jiaotong University, Xi’an, China; 2 School of Life Science and Technology, Xidian University, Xi’an, China; 3 School of Optoelectronic Engineering, Henan Normal University, Xinxiang, China

**Keywords:** ciprofol, consciousness, EEG, frequency-specific analysis, microstate analysis

## Abstract

**Background:**

Ciprofol is a novel GABAergic intravenous anesthetic with a rapid onset and favorable safety profile; however, the neural mechanisms underlying the induction and reversal of unconsciousness remain unclear. Electroencephalogram (EEG) microstates provide millisecond-scale markers of large-scale brain network dynamics and may reveal frequency-specific signatures of ciprofol’ s effects on consciousness.

**Methods:**

Sixty-channel EEG was recorded during wakefulness, ciprofol-induced loss of consciousness (LOC), and recovery of consciousness (ROC). Power spectra were computed across the delta, theta, alpha, beta, and gamma bands. Microstate analysis was performed separately for each band, yielding seven microstates (A–G). Duration, coverage, and occurrence were compared across conscious states using one-way analysis of variance (ANOVA) with Bonferroni correction. Support vector machine classifiers were trained on broadband and frequency-specific microstate features to distinguish the conscious states.

**Results:**

Ciprofol increased delta–alpha power during LOC and maintained elevated beta–gamma activity during early ROC. Microstate parameters showed clear state-dependent changes across frequencies: microstate D decreased in the delta–theta bands during LOC; alpha-band microstates displayed reduced duration and increased occurrence; and microstates A, C, and E in the beta–gamma bands showed significant alterations in occurrence, coverage, or explained variance. Classification improved with sub-band features, with alpha band achieving the highest accuracy (0.841) compared with broadband features (0.754). Moreover, classification models integrating features of all sub-frequency bands yielded superior performance in distinguishing conscious states, achieving an accuracy of 0.971.

**Conclusion:**

Ciprofol induces a distinct frequency-specific reorganization of cortical microstates, revealing multiscale network signatures of unconsciousness and recovery. Frequency-resolved microstate metrics may serve as sensitive markers for characterizing anesthetic-induced brain state transitions.

## 1 Introduction

Ciprofol (HSK3486) is a novel intravenous anesthetic developed as an alternative to propofol, which is one of the most widely used agents for clinical anesthesia (Lu et al., 2023). Although structurally similar to propofol, it is distinguished by the addition of a cyclopropyl group; ciprofol acts as a potent *γ*-aminobutyric acid-A (GABA_A_) receptor agonist with higher receptor affinity, enabling rapid induction, effective sedation at lower doses, and accelerated metabolic clearance (Qin et al., 2017; Liao et al., 2022). Its limited effects on respiratory and cardiovascular functions, together with markedly reduced injection pain relative to propofol, further underscore its clinical promise (Zhou et al., 2025). A growing body of clinical evidence supports the efficacy and safety of ciprofol, positioning it as a viable and potentially advantageous alternative to propofol in diverse anesthetic settings (Luo et al., 2022; Liu et al., 2023; Gan et al., 2024; Ni et al., 2025). Despite these advances, the neural mechanisms by which ciprofol modulates consciousness remain unclear, potentially limiting its broader clinical application.

Electroencephalography (EEG) is a widely adopted approach for probing anesthesia-related changes in consciousness owing to its noninvasiveness, high temporal resolution, and sensitivity to large-scale neural dynamics (Hagihira, 2015; Purdon et al., 2015b; Kreuzer, 2017). Although ciprofol has been extensively evaluated for its clinical efficacy and safety as an alternative to propofol, EEG investigations remain scarce. A recent study examined the neural oscillatory characteristics during ciprofol-induced general anesthesia using low-density frontal EEG, reporting spectral changes similar to those observed with propofol (Zhu et al., 2026). However, conventional spectral analysis primarily captures changes in oscillatory power and provides limited insight into the spatiotemporal organization of large-scale brain network dynamics.

EEG microstates—brief, quasi-stable topographical patterns often described as “atoms of thought”—provide a powerful window into the rapid transition of large-scale brain networks (Khanna et al., 2015; Michel and Koenig, 2018; Tarailis et al., 2024). Accumulating evidence shows that EEG microstates are strongly modulated by fluctuations in levels of consciousness across physiological, pathological, and pharmacological states (Brodbeck et al., 2012; Bréchet and Michel, 2022; Toplutaş et al., 2023; Asha et al., 2024). Under propofol anesthesia, microstate dynamics follow a characteristic U-shaped trajectory, with increased temporal complexity and occurrence during moderate sedation, followed by reduced complexity, prolonged duration, and diminished metastability at deeper levels of unconsciousness (Wang et al., 2021; Artoni et al., 2022; Liu et al., 2022; Lapointe et al., 2023). These findings highlight the potential of microstate analysis as a sensitive tool for tracking drug-induced changes in consciousness. Therefore, we hypothesized that characterizing EEG microstate dynamics under ciprofol anesthesia would provide critical clues to the neural mechanisms mediating ciprofol-induced loss of consciousness (LOC).

EEG microstates are conventionally analyzed within broad frequency ranges, such as 2–20 Hz or 0.5–45 Hz (Koenig et al., 2002; Milz et al., 2017; Shi et al., 2020). Ciprofol, like propofol, acts primarily through potentiation of GABA_A_ receptors in the central nervous system, producing sedation and hypnosis (Qin et al., 2017). During propofol-induced LOC, EEG exhibits characteristic power spectrum changes, including increased slow-wave activity (<1 Hz), dissipation of spatially coherent occipital alpha oscillations, and alpha anteriorization with prominent frontal alpha power (Ní Mhuircheartaigh et al., 2013; Purdon et al., 2013). Given their pharmacological similarities, ciprofol is expected to elicit comparable spectral signatures. However, emerging evidence indicates that broadband microstate analysis may overlook band-specific neural correlates critical for distinguishing conscious states. For example, EEG signals excluding the delta band have been reported to demonstrate superior sensitivity in distinguishing conscious states compared with the delta band alone (Zhang et al., 2025). Hence, frequency-specific EEG microstate dynamics may offer greater sensitivity for tracking state transitions than conventional broadband approaches.

The aim of this study was to investigate the frequency-specific EEG microstate dynamics during ciprofol-induced alterations in consciousness. High-density 60-channel EEG recordings were acquired during ciprofol anesthesia to characterize power spectral changes across key frequency bands (delta: 1–4 Hz, theta: 4–8 Hz, alpha: 8–15 Hz, beta: 15–30 Hz, and gamma: 30–45 Hz) associated with transitions into and out of unconsciousness. Microstate analysis was performed within each frequency band to assess spatiotemporal properties, including duration, occurrence, coverage, and explained variance. These microstate features were subsequently used as inputs for machine-learning classifiers to distinguish among wakefulness, unconsciousness, and recovery states. By integrating spectral and microstate analyses within a frequency-resolved framework, the aim of this study was to provide deeper insights into the neural correlates of ciprofol anesthesia.

## 2 Materials and methods

### 2.1 Participants

This study was approved by the Ethics Review Board of the First Affiliated Hospital of Xi’an Jiaotong University (Clinical Trial Registration: ChiCTR2400094266) and conducted in accordance with the principles of the Declaration of Helsinki. Written informed consent was obtained from all participants prior to enrollment. A total of 24 healthy male volunteers (age: 30.17 ± 4.94 years) were recruited. All participants were free of medication and reported no known allergies to ciprofol ingredients (i.e., soybean oil, glycerol, triglycerides, yolk lecithin, sodium oleate, and sodium hydroxide). The exclusion criteria included a history of neurological or psychiatric disorders, participation in pharmacological clinical trials within the preceding 3 months, and a body mass index greater than 28 kg/m^2^. Participants were instructed to fast for approximately 8 h before ciprofol administration.

### 2.2 Anesthesia protocol

Anesthesia induction and monitoring were performed by two senior anesthesiologists. Upon arrival in the operating room, venous access was established in the upper extremities, and Ringer’s lactate solution was administered at a rate of 10 mL/kg/h. Throughout the experiment, the participants remained in a reclined supine position while continuous EEG recordings were obtained. Vital signs, including heart rate, noninvasive blood pressure, and oxygen saturation, were continuously monitored using standard intraoperative monitoring equipment (Philips MP50, Boeblingen, Germany). A bispectral index (BIS) monitor (Covidien, Mansfield, MA, United States) was used as a standard clinical monitor to ensure participant safety.

Before ciprofol administration, participants were instructed to remain relaxed and awake for 5 min to establish baseline recordings. An induction dose of ciprofol (Haisco, Xingcheng, Liaoning, China) at 0.4 mg/kg was then administered intravenously for 30 s, followed by a maintenance infusion of 0.8 mg/kg/h for 5 min. Subsequently, the infusion was discontinued. Participants were considered awake upon their first correct response to the verbal command “open your eyes.” After EEG data were recorded for an additional 3 min, the experiment was terminated, and the participants were transferred to the anesthesia recovery room to await full awakening. The ciprofol administration protocol is shown in Supplementary Figure S1.

Three states of consciousness were examined: wakefulness, ciprofol-induced LOC, and recovery of consciousness (ROC). In line with current clinical and research standards in anesthesia EEG studies, the three consciousness states were defined based on behavioral responsiveness to a standardized verbal command (“open your eyes”), which remains the gold-standard operational definition of loss and return of consciousness (Ní Mhuircheartaigh et al., 2013; Purdon et al., 2013; Zhang et al., 2023). The BIS was used as a clinical safety measure rather than as a primary or secondary study outcome; therefore, no formal statistical tests were performed on the BIS values. To ensure completeness, the mean BIS values were calculated for each conscious state. The results showed that during wakefulness, the mean BIS value was 88.6 (median, range 78.7–96.6). The mean BIS value during LOC decreased to 56.7 (median, range 47.7–70.8) and subsequently recovered to 77.6 (median, range 70.3–90.1) during ROC.

### 2.3 EEG data acquisition and preprocessing

EEG data were acquired using a 60-channel SynAmps RT system (NeuroScan, Germany) with reference electrodes placed on the left and right mastoids. During wakefulness EEG acquisition, participants were instructed to remain relaxed with their eyes closed. Signals were sampled at 1,000 Hz, and electrode impedance was maintained below 5 kΩ throughout the recording to ensure high data quality.

Preprocessing was performed using EEGLAB (version 2024). Raw EEG signals were downsampled to 250 Hz and bandpass filtered between 1 and 45 Hz using a zero-phase, fifth-order Butterworth filter (*filtfilt.m* function in MATLAB). Channels exhibiting excessive noise or artifacts were identified through visual inspection and replaced using spherical spline interpolation. Next, an Infomax independent component analysis (ICA) algorithm with default parameters (*pop_runica.m* function) was applied to the continuous EEG data using EEGLAB routines. Components corresponding to ocular, muscular, and cardiac artifacts were automatically identified and rejected using the MARA toolbox (Winkler et al., 2011). The cleaned EEG signals were then re-referenced to a common average.

**Figure 1:**
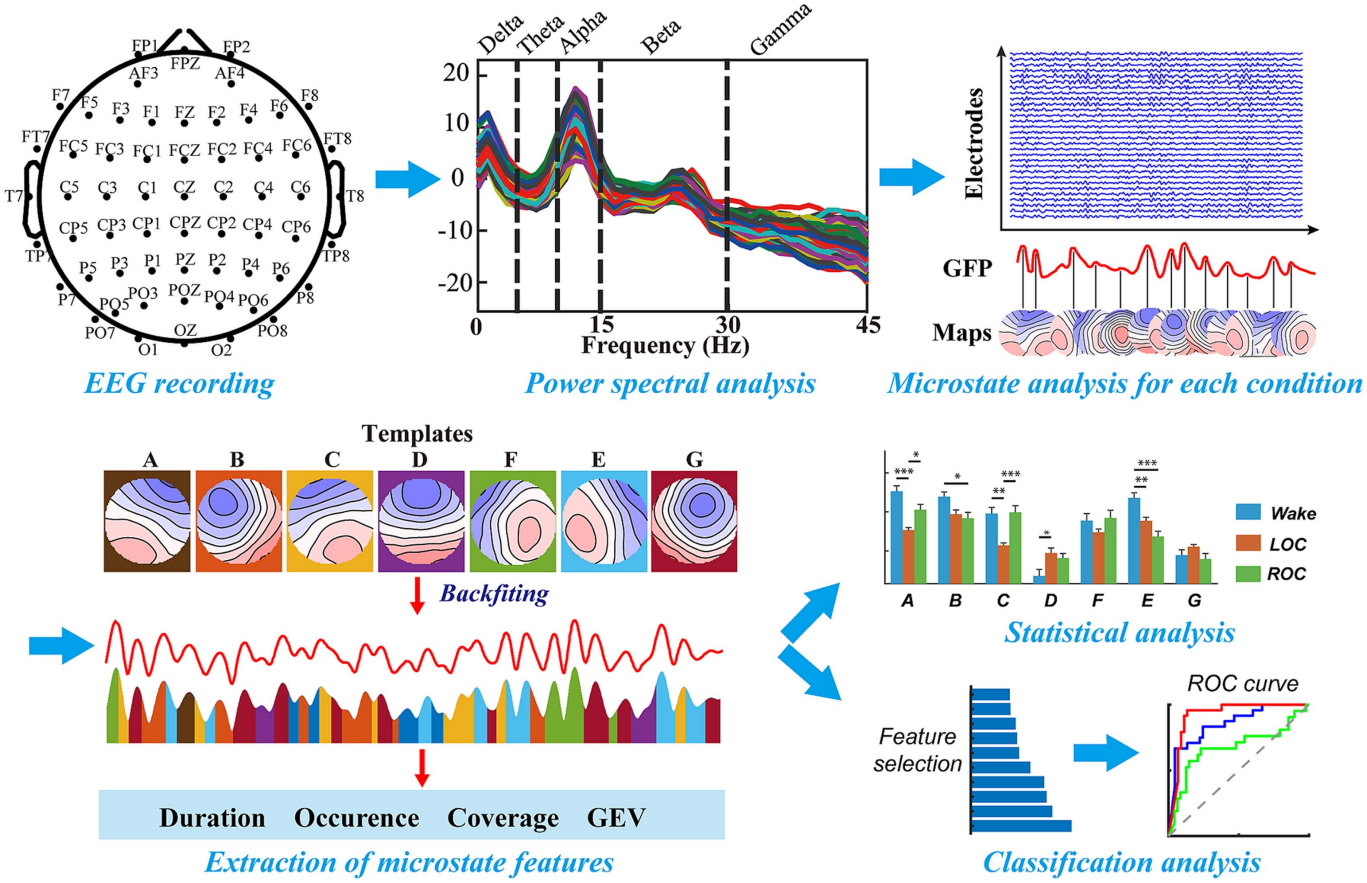
Schematic overview of the EEG processing and analysis pipeline for frequency-specific microstate characterization of ciprofol-induced alterations in consciousness. High-density 60-channel EEG recordings were preprocessed (bandpass filtered 1–45 Hz, artifact rejection via ICA, common-average re-referencing) and segmented into wakefulness, loss of consciousness (LOC), and recovery of consciousness (ROC) epochs. Power spectral density was computed using the multitaper method (2-s sliding windows) across delta, theta, alpha, beta, and gamma bands. Within each band, modified *K*-means clustering identified seven microstate topographies (A–G), which were back-fitted to global field power peaks to extract temporal features (mean duration, occurrence, coverage, and individual explained variance). These features were statistically compared across states (repeated-measures ANOVA with Bonferroni correction) and used for machine-learning classification (SVM with hybrid NCA + MRMR feature selection, top-10 features) (see Materials and Methods 2.3–2.7).

For microstate analysis, continuous artifact-free EEG segments were extracted for each consciousness state as follows: wakefulness (pre-induction baseline, 5 min), maintenance infusion stage (LOC, 5 min), and after behavioral recovery (ROC, 3 min). Owing to poor signal quality, the ROC-phase data from three participants were excluded. Consequently, EEG data for both wakefulness and LOC were available for all 24 participants, whereas ROC data were available for only 21 participants. A schematic overview of the EEG preprocessing and analysis pipeline is shown in Figure 1.

### 2.4 Spectral analysis

Power spectral analysis was performed using the multitaper method implemented in the MATLAB-compatible Chronux toolbox (Bokil et al., 2010). Spectral estimates were computed within the 1–45 Hz frequency range to maintain consistency with subsequent microstate analyses. The analysis employed a 2-s sliding window with 0.5-s overlap, a time–bandwidth product of 3, and five Slepian tapers. The power spectral density (PSD) was calculated for five canonical frequency bands: delta (1–4 Hz), theta (4–8 Hz), alpha (8–15 Hz), beta (15–30 Hz), and gamma (30–45 Hz). For each participant, the PSD values were first averaged across frequency bins within each band and subsequently averaged across all channels to derive the mean spectral power for the wakefulness, LOC, and ROC conditions. Topographical maps of band-limited spectral power during LOC and ROC were generated after wakefulness normalization to visualize the spatial distribution of ciprofol-induced spectral changes.

Multitaper estimation provides lower-variance PSD estimates without requiring excessively long windows and is therefore advantageous for short data segments (Babadi and Brown, 2014). The window length selection of 2-s provides a frequency resolution of 0.5 Hz, which is sufficient to clearly separate the canonical EEG frequency bands of interest with minimal spectral leakage between adjacent bands. Using multiple orthogonal tapers (taper = 5), the multitaper spectral analysis effectively increases the degrees of freedom, providing stable and reliable power estimates even in the low-frequency band. Moreover, a 2-s window is commonly used in anesthesia EEG studies that require simultaneous resolution of slow oscillations and faster dynamics during state transitions (Purdon et al., 2013; Akeju et al., 2014, 2016).

### 2.5 Microstate analysis

The microstate analysis was performed using the MICROSTATELAB toolbox (Nagabhushan Kalburgi et al., 2024). Prior to microstate extraction, the preprocessed EEG data were filtered into six distinct frequency bands: broadband (1–45 Hz), delta (1–4 Hz), theta (4–8 Hz), alpha (8–15 Hz), beta (15–30 Hz), and gamma (30–45 Hz). Microstate analysis was conducted independently for each frequency band.

For each band-specific EEG signal, the global field power (GFP), defined as the standard deviation of scalp potentials across all electrodes at each time point, was first calculated. Topographies at the GFP peaks were selected to represent momentarily stable scalp potential configurations owing to their high signal-to-noise ratio. These maps were subjected to a modified k-means clustering algorithm to derive the individual-level microstate classes. The optimal number of microstate classes (*k* = 2–8) for each consciousness state and frequency band was determined using the global explained variance (GEV) and cross-validation (CV) criteria, with 20 clustering iterations per model. GEV was computed as the sum of the variance explained by each microstate class, and the explained variance for each class was expressed as a percentage of the total variance.

Microstates were first computed at the individual level for each condition (i.e., individual-level clustering). Condition-level mean maps were generated by averaging the microstate maps across participants within each state. Grand-mean microstate maps were subsequently produced by averaging condition-level maps across all states and ordered according to normative template microstate maps (Custo et al., 2017). Individual- and condition-level maps were sorted according to the ordered grand-mean templates to ensure consistent microstate labeling across participants and conditions, which is a prerequisite for valid between-subject and between-state comparisons.

The sorted grand-mean microstate maps were then back-fitted to each participant’s continuous EEG data. At each time point, a microstate label corresponding to the map with the highest spatial correlation was assigned. Microstate labels between adjacent GFP peaks were interpolated, with microstates beginning and ending halfway between successive peaks. Microstate fragments that were truncated at the start or end of an epoch were excluded.

From the resulting microstate time series, standard temporal parameters were quantified for each microstate class and frequency band across three consciousness states: duration (Dur), occurrence (Occ), and coverage (Cov). Duration was defined as the mean uninterrupted time during which a map remained assigned to the same microstate class, reflecting the temporal stability of its neural generators. Occurrence was computed as the average number of appearances per second, indexing the propensity of a microstate class to be activated. Coverage was defined as the percentage of total analysis time occupied by each microstate class, reflecting its relative contribution to ongoing neural activity (Khanna et al., 2015). Additionally, the individual explained variance (IEV) was extracted to quantify each microstate class’s contribution to the total variance within each frequency band and consciousness state.

### 2.6 Statistical analysis

Statistical analyses were performed on the mean power spectra and microstate features derived from each frequency band. The normality of all microstate parameters was formally assessed separately for each frequency band and consciousness state using the Lilliefors test (significance level = 0.05). Approximately 78% of the parameters satisfied the normality assumption (*p* > 0.05). Given the robustness of repeated-measures ANOVA to moderate violations of normality with balanced sample sizes (Blanca et al., 2023), parametric ANOVA was used for all comparisons. The Bonferroni correction was applied separately within each frequency band to account for multiple pairwise comparisons. To enable within-subject statistical testing, only data from the 21 participants who had valid EEG recordings for all three states (wakefulness, LOC, and ROC) were included in the analysis.

### 2.7 Classification analysis

A linear support vector machine (SVM) classifier was used to discriminate among the three consciousness states (wakefulness, LOC, and ROC) based on the microstate parameters extracted from each frequency band. In total, eight classifiers were trained. Six classifiers were built separately for each frequency band (broadband, delta, theta, alpha, beta, and gamma) using 28 original microstate features (7 microstates × 4 parameters: mean duration, occurrence, coverage, and GEV). Two integrative models were also constructed: one using microstate features from all sub-bands (delta, theta, alpha, beta, and gamma), yielding 140 original features (5 bands × 7 microstates × 4 parameters), and the other combining broadband and all sub-band features, yielding 168 original features (6 bands × 7 microstates × 4 parameters). These microstate parameters were derived from 60-channel EEG data via modified k-means clustering on GFP peaks, and the resulting microstate features (rather than the raw multichannel time series) were used as inputs for feature selection and classification. All models were trained and evaluated using 63 complete-case samples (21 participants × 3 consciousness states). All features were standardized before model training by subtracting the mean and scaling to unit variance.

For each classifier, feature selection was performed to identify the most informative microstate features for consciousness-state classification. A hybrid scoring strategy combining neighborhood component analysis (NCA) and minimum redundancy maximum relevance (MRMR) was employed. The NCA weights were computed using the MATLAB function *fscnca.m*, which learns a feature-weighting metric by maximizing the leave-one-out classification accuracy of a stochastic nearest-neighbor model (Raghu and Sriraam, 2018). MRMR scores were obtained using *fscmrmr.m*, which ranks features by maximizing mutual information with class labels while minimizing redundancy among the selected features (Radovic et al., 2017). Both the NCA and MRMR scores were min–max normalized and summed to generate a composite importance score for each feature. The top 10 features with the highest composite scores were selected as the final inputs for the SVM classifiers.

The SVM classifier was selected as the primary classifier because of its strong performance with small-sample, high-dimensional neurophysiological data and its demonstrated effectiveness in previous EEG microstate classification studies during anesthesia (Wang et al., 2021; Zhang et al., 2025). Classifier performance was evaluated using leave-one-out cross-validation (LOOCV), which was appropriate for the sample size of this study. In each iteration, the classifier was trained on data from all but one participant, and the held-out participant was used for testing. To address concerns regarding potential optimism bias associated with LOOCV in small samples, classifier performance was also evaluated using stratified five-fold cross-validation. Moreover, a Random Forest (number of trees = 100) was included as a complementary model for comparative evaluation. All evaluation strategies used the same selected features and hyperparameter optimizations. Performance metrics, including overall accuracy, class-wise F1 scores, receiver operating characteristic curves, and the corresponding areas under the curve (AUCs), were computed by aggregating results across all folds.

## 3 Results

### 3.1 Spectral and topographic characteristics during ciprofol anesthesia

**Figure 2:**
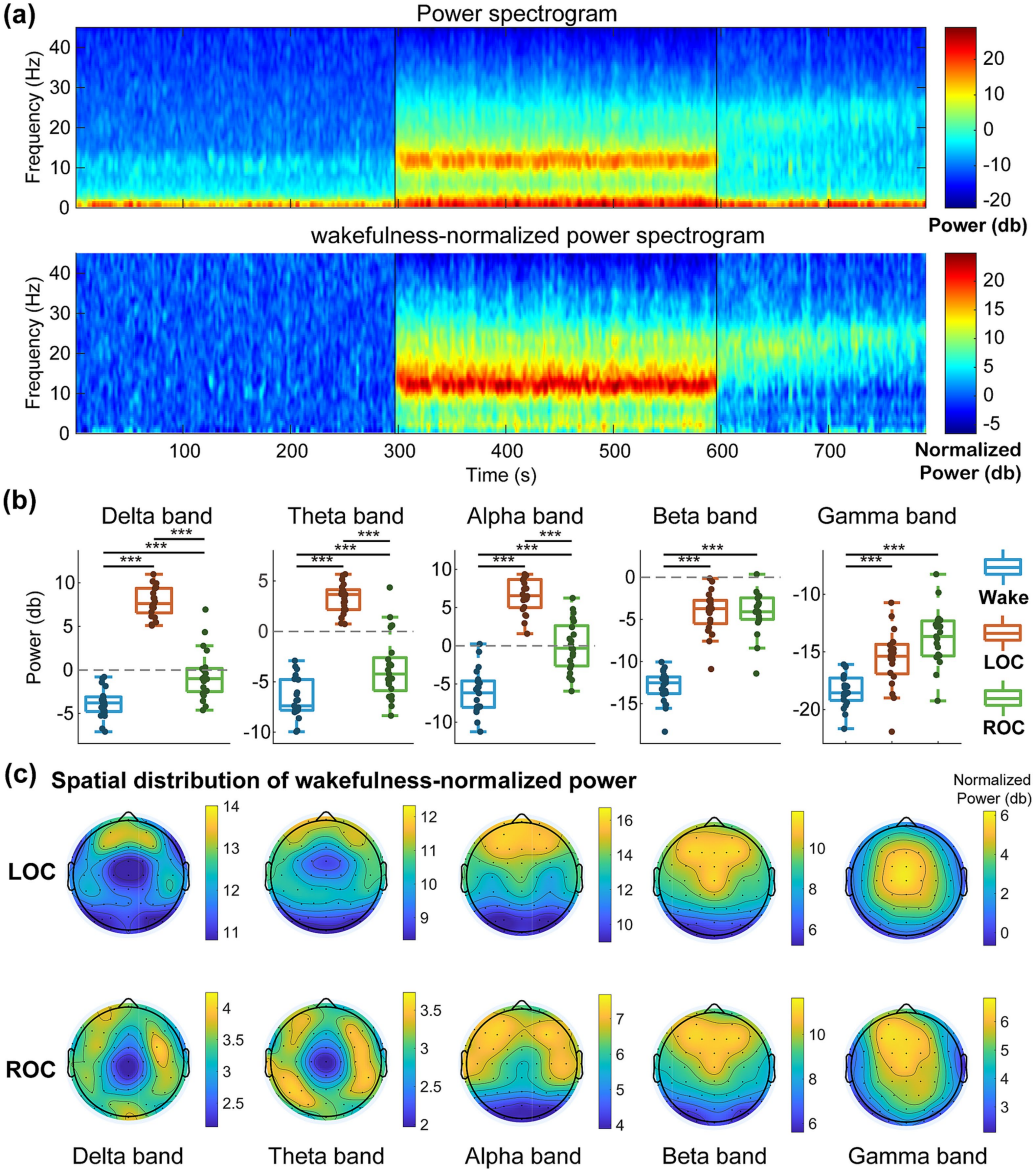
Spectral power alterations induced by ciprofol anesthesia across wakefulness, loss of consciousness (LOC), and recovery of consciousness (ROC). Power spectral density was estimated using the multitaper method (2-s sliding windows, 250 Hz sampling rate) and normalized to the wakefulness baseline for visualization. **(a)** Representative time–frequency spectrograms from a single participant (frontal region): Raw power (top) and wakefulness-normalized power (bottom), showing the characteristic increase in alpha-band (8–13 Hz) power during LOC with partial recovery at ROC. **(b)** Group-level mean power (*n* = 21 participants with complete data) in the delta (1–4 Hz), theta (4–8 Hz), alpha (8–13 Hz), beta (13–30 Hz), and gamma (30–45 Hz) bands. Boxplots display median, interquartile range, and individual data points; ***p < 0.001 (repeated-measures ANOVA with Bonferroni-corrected *post-hoc* tests). **(c)** Topographic maps of wakefulness-normalized power during LOC and ROC, highlighting alpha anteriorization and frontal power enhancement during unconsciousness. These frequency-specific spectral signatures demonstrate that ciprofol produces propofol-like EEG changes and provide the foundation for subsequent frequency-resolved microstate analysis (see Results 3.1 and Discussion 4.1).

As shown in Figure 2a, the time–frequency spectrogram from a representative frontal electrode demonstrated a progressive increase in broadband power as participants transitioned into unconsciousness, with particularly prominent enhancements in low-frequency (delta) and alpha activity. During recovery, both delta and alpha power partially returned toward wakefulness levels, whereas activity in the higher-frequency beta and gamma bands remained elevated relative to wakefulness.

These observations were confirmed by group-level spectral analysis (Figure 2b), which revealed significant differences in power across conscious states for all frequency bands (delta: *F*_2, 40_ = 169.33, *p* < 0.001; theta: *F*_2, 40_ = 101.89, *p* < 0.001; alpha: *F*_2, 40_ = 103.55, *p* < 0.001; beta: *F*_2, 40_ = 101.03, *p* < 0.001; gamma: *F*_2, 40_ = 24.66, *p* < 0.001). Specifically, delta, theta, and alpha power increased significantly during LOC (*p* < 0.001) and subsequently decreased during ROC (*p* < 0.001), although none returned fully to wakefulness levels. In contrast, beta and gamma power increased markedly during LOC (*p* < 0.001) and remained elevated during ROC.

The spatial distributions of wakefulness-normalized power during LOC and ROC (Figure 2c) further illustrated these effects. Increases in delta, theta, and alpha activity during LOC were maximal over the frontal cortex, whereas beta and gamma enhancements were distributed across the frontal and central regions.

### 3.2 Optimal number of microstates and microstate topographies

**Figure 3:**
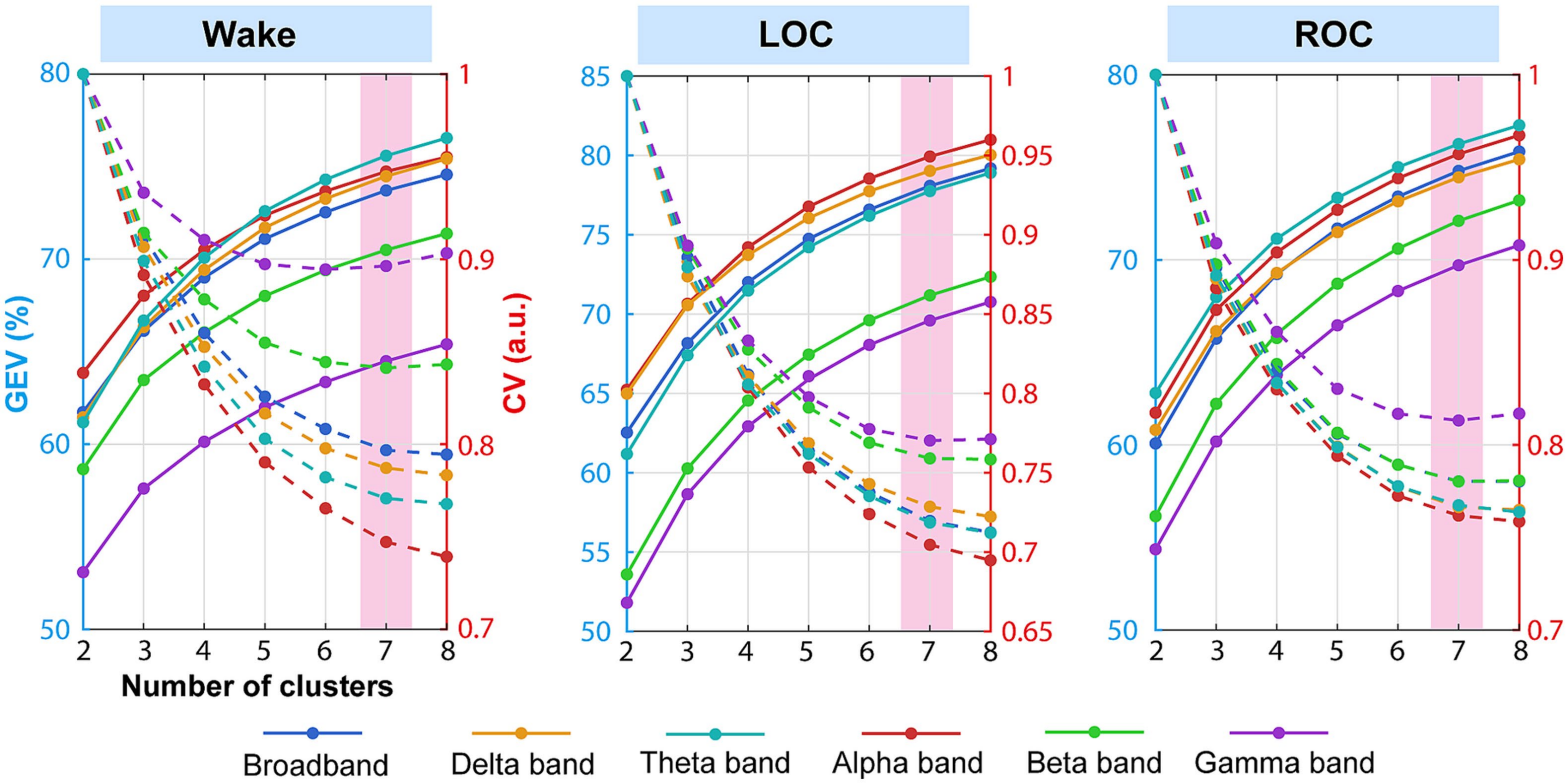
Determination of the optimal number of microstates (*K* = 7) across frequency bands and consciousness states under ciprofol anesthesia. Global explained variance (GEV, solid lines) and cross-validation prediction error (CV, dashed lines) were plotted against cluster number (*K* = 2–8) for broadband and each frequency-specific band (delta, theta, alpha, beta, and gamma) separately in wakefulness, loss of consciousness (LOC), and recovery of consciousness (ROC). All 18 curves exhibited a clear elbow at *K* = 7. This consistent elbow across all bands and states justified the uniform selection of seven microstates, enabling direct comparability of temporal parameters in subsequent frequency-specific analyses (see Results 3.2).

We determined the optimal number of microstate templates for each frequency band and conscious state using the standard dual-criterion approach by maximizing the GEV while minimizing the CV prediction error, testing cluster numbers from two to eight. GEV–CV curves were generated separately for each frequency band and consciousness state. As illustrated in Figure 3, GEV increased monotonically, whereas the CV criterion decreased progressively as the number of clusters increased. Under most conditions, the curves exhibited a clear elbow at *k* = 7. Compared with *k* = 6, selecting *k* = 7 produced a median GEV increase of 1.99% (range 1.55–2.31%) and a median CV error reduction of 1.85% (range −0.24–2.93%) across all conditions. Increasing the number of microstates to *k* = 8 yielded only marginal improvements (median GEV increase 1.46%, range 1.10–1.72%; median CV reduction 0.72%, range of −0.92–1.39%), indicating diminishing returns and increased risk of overfitting. Therefore, seven microstates were retained for all subsequent analyses to ensure consistency and comparability across bands and states.

**Figure 4:**
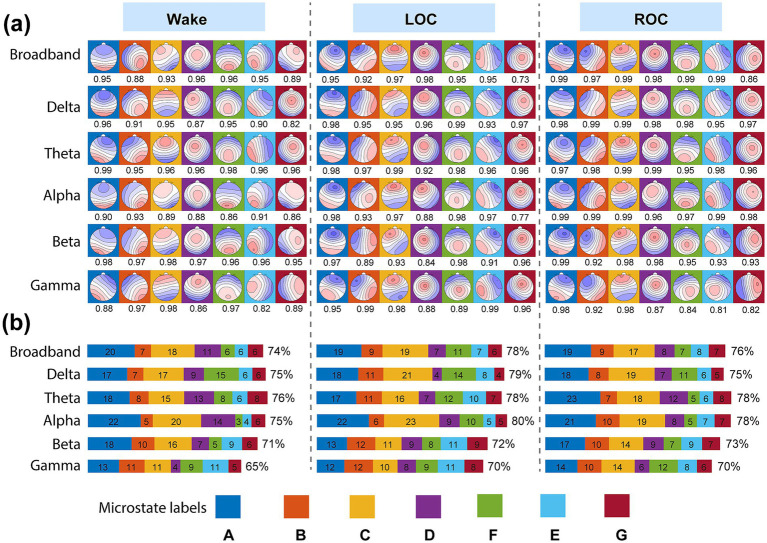
Identification and validation of seven microstate topographies across frequency bands and consciousness states under ciprofol anesthesia. **(a)** Grand-mean spatial maps of the seven microstates (A–G) identified via modified K-means clustering on global field power peaks for each frequency band (broadband, delta, theta, alpha, beta, gamma) and consciousness state (wakefulness, LOC, ROC). Maps were sorted to best match canonical templates (Custo et al., 2017); spatial correlation coefficients with the corresponding template are displayed below each map. **(b)** Global explained variance (GEV) and individual explained variance (IEV) contributed by each microstate class for every band and state. The consistently high GEV (>80% in most cases) and strong spatial correlations across all conditions confirmed that seven microstates provide a robust and stable representation of brain dynamics (see Results 3.2 and Discussion 4.2).

As shown in Figure 4a, the microstate maps were labeled according to established canonical templates (Custo et al., 2017) as follows: left posterior-to-right frontal (A), right posterior-to-left frontal (B), anterior–posterior (C), fronto-central maximum (D), left–right orientation (E), occipito-central maximum (F), and central–posterior maximum (G). Spatial correlations between the extracted microstates and their corresponding templates consistently exceeded 0.85 across conditions, confirming the robust reproducibility of microstate topographies under ciprofol anesthesia.

Figure 4b presents the GEV and IEV of the microstate classes across all conditions. The seven microstate classes collectively accounted for more than 70% of the variance in GFP peaks in all cases except the gamma band during wakefulness. The alpha band exhibited the highest mean explained variance (80% during LOC), whereas the gamma band exhibited the lowest (65% during wakefulness). Furthermore, we compared the IEV of the microstate classes across the wakefulness, LOC, and ROC states. The results are presented in Supplementary Figure S2; Supplementary Table S1. Microstates A and C exhibited the highest explained variance across most frequency bands. In all bands except gamma, the explained variance of microstate D decreased, and that of microstate F increased during LOC, with the opposite pattern observed during ROC. For microstate E, the explained variance increased in the broadband and alpha bands but decreased in the gamma band during ROC compared with wakefulness.

### 3.3 Frequency-specific EEG microstate dynamics across consciousness states

**Figure 5:**
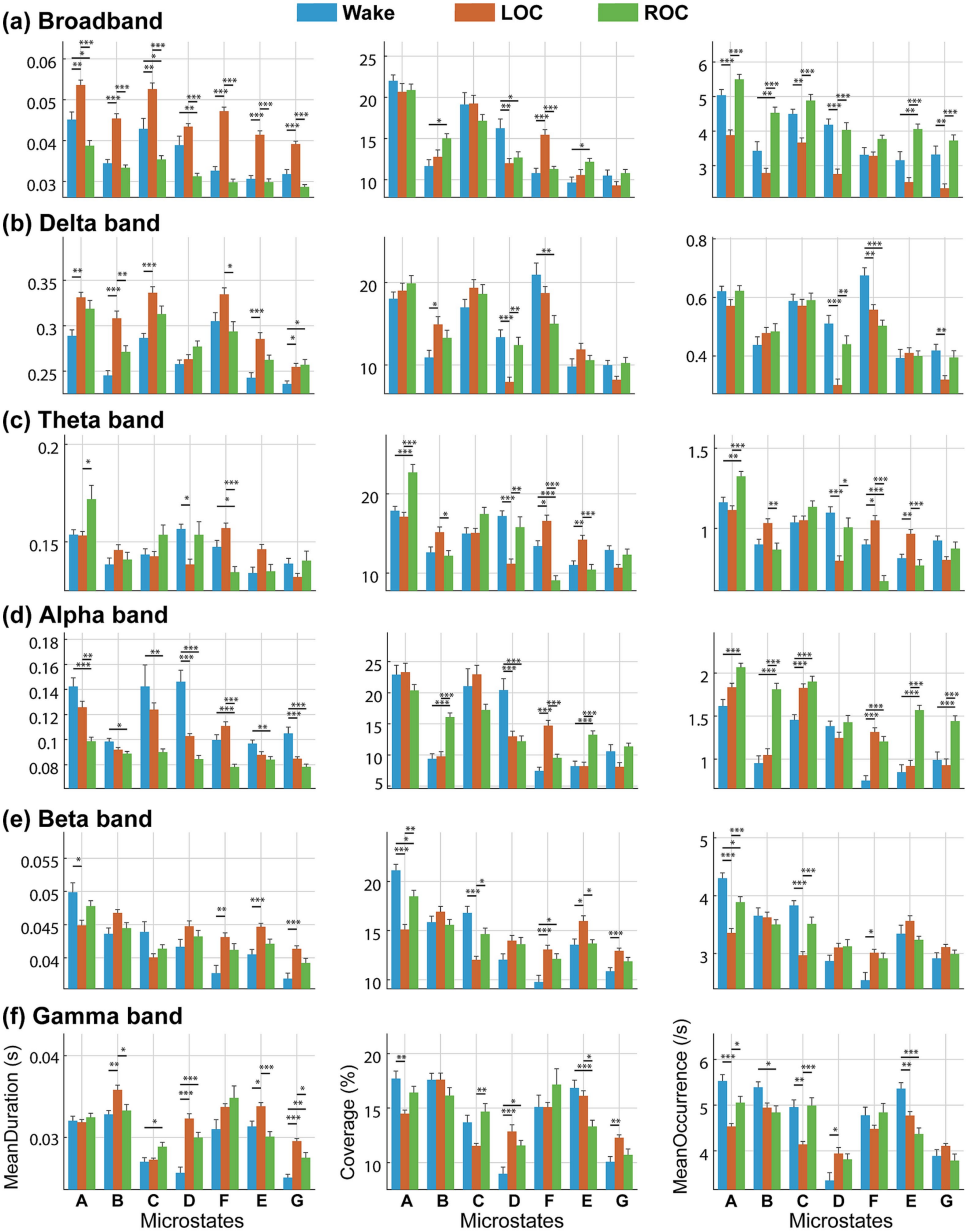
Frequency-specific changes in EEG microstate parameters across wakefulness, loss of consciousness (LOC), and recovery of consciousness (ROC) under ciprofol anesthesia. Group-level comparisons (*n* = 21 participants with complete data) of mean duration, occurrence, and coverage for seven microstates (A–G) in **(a)** Broadband (1–45 Hz), **(b)** Delta (1–4 Hz), **(c)** Theta (4–8 Hz), **(d)** Alpha (8–13 Hz), **(e)** Beta (13–30 Hz), and **(f)** Gamma (30–45 Hz) bands. Data are presented as mean ± SEM. Statistical comparisons were performed using one-way repeated-measures ANOVA with Bonferroni-corrected post-hoc tests (*p < 0.05, **p < 0.01, ***p < 0.001). Key patterns include consistent prolongation of duration and reduction in occurrence during LOC (with recovery at ROC) in broadband and low-frequency bands, contrasted with progressive duration decreases and occurrence increases in the alpha band. These frequency-dependent reorganizations demonstrate that frequency-specific microstate features provide enhanced sensitivity for tracking consciousness transitions compared with conventional broadband analysis (see Results 3.3 and Discussion 4.3).

We estimated and compared three microstate features—mean duration, coverage, and mean occurrence—to characterize frequency-specific EEG microstate dynamics across consciousness states. Detailed statistical results are summarized in Figure 5 and Supplementary Tables S2–S7.

In the broadband EEG (Figure 5a), nearly all microstate features exhibited significant state-dependent differences (*p* < 0.05). Compared with wakefulness, the LOC state was marked by a generalized increase in mean duration and a decrease in mean occurrence across all microstates (A–G), with both features returning toward baseline during ROC. Coverage changes were moderate but remained significant for microstates B, D, F, and E (*p* < 0.05), with microstate F showing the largest increase during LOC.

As shown in Figure 5b, the delta band showed prolonged durations during LOC for most microstates (*p* < 0.05). Differences in coverage across consciousness states were significant for microstates B, D, and F (*p* < 0.05), with microstate D showing the largest decrease during LOC, followed by a significant rebound during ROC (*p* < 0.01). Mean occurrence showed fewer but consistent effects, with microstates D, F, and G displaying lower occurrence at LOC (*p* < 0.01). The mean occurrence of microstates D and G recovered during the ROC state, whereas microstate F remained lower than during wakefulness (*p* < 0.001).

Distinct microstate-specific patterns were observed in the theta band (Figure 5c). Microstate A showed no significant changes in any parameter during LOC (*p* > 0.05); however, all three features increased significantly during ROC (*p* < 0.05). In contrast, microstate D showed significant reductions in duration, coverage, and occurrence during LOC (*p* < 0.05), followed by recovery to wakefulness at ROC. Microstates F and E displayed the opposite pattern, with significant increases across all three parameters during LOC (*p* < 0.05) and returning to wakefulness levels during ROC.

For the alpha band (Figure 5d), most microstates exhibited a consistent decrease in mean duration across consciousness states (*p* < 0.05), with values declining progressively from wakefulness to LOC and further during ROC. However, the mean occurrence showed a monotonic increase across states. Coverage showed heterogeneous effects. Microstates B and E showed no differences between wakefulness and LOC (*p* > 0.05) but increased significantly at ROC (*p* < 0.001). Microstate D demonstrated a marked reduction in coverage during LOC (*p* < 0.001), which persisted during ROC (*p* > 0.05). In contrast, microstate F showed increased coverage during LOC (*p* < 0.001), followed by a return to baseline during ROC.

In the beta band (Figure 5e), microstates A and C exhibited significant reductions in all three parameters during LOC (*p* < 0.05), with recovery during ROC. In contrast, microstates F, E, and G showed significant increases in mean duration, coverage, and occurrence during LOC (*p* < 0.05), and these values returned to near-wakefulness levels during ROC.

In the gamma band (Figure 5f), multiple microstates exhibited significant state effects. Microstates A and C showed reduced coverage and occurrence during LOC (*p* < 0.05), followed by partial recovery during ROC. Microstate E exhibited a continuous reduction in both coverage and occurrence from wakefulness through LOC to ROC (*p* < 0.05) with no evidence of recovery. In contrast, microstate D exhibited significant increases in duration, coverage, and occurrence during LOC (*p* < 0.05) and remained elevated during ROC.

### 3.4 Classification of consciousness states using microstate features

**Figure 6:**
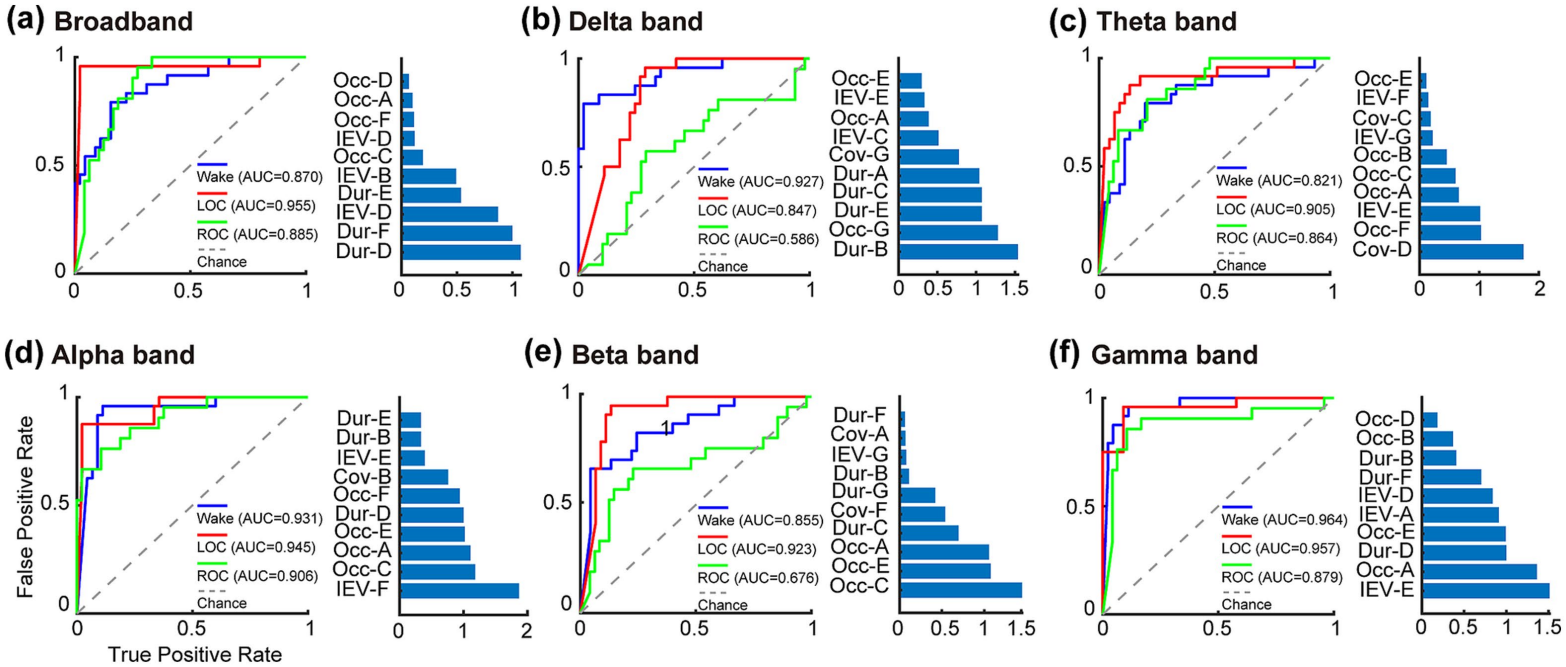
Classification performance of SVM models using frequency-specific EEG microstate features. Receiver operating characteristic curves and corresponding top-10 features (ranked by composite NCA + MRMR importance score) for **(a)** broadband, **(b)** delta, **(c)** theta, **(d)** alpha, **(e)** beta, and **(f)** gamma bands. Models were trained on the top-10 microstate features extracted from 63 complete-case samples (21 participants × 3 states) using leave-one-out cross-validation. Area under the curve (AUC) values are shown for each state (wakefulness, LOC, ROC). The superior performance of alpha and gamma bands supported that the frequency-specific microstate features provided enhanced performance for distinguishing consciousness states compared with broadband analysis (see Results 3.4 and Discussion 4.4).

SVM classifiers were trained to discriminate among wakefulness, LOC, and ROC using microstate features (mean duration, coverage, mean occurrence, and IEV) derived from each frequency band. The overall classification accuracy, macro-F1 score, and per-class F1 scores are summarized in Table 1. Using broadband microstate features, the classifier achieved an overall accuracy of 0.754 with a macro-F1 score of 0.752. As shown in Figure 6a, the most influential predictors included the mean durations of microstates D, E, and F and the explained variance of microstate D (importance scores > 0.5).

**Table 1:** Classification performance of SVM models using frequency-specific EEG microstate features.

Features	Accuracy	Micro-F1	F1-wake	F1-LOC	F1-ROC
Broadband	0.754	0.752	0.652	0.936	0.667
Delta band	0.623	0.618	0.792	0.596	0.465
Theta band	0.739	0.735	0.731	0.809	0.667
Alpha band	0.841	0.840	0.857	0.889	0.773
Beta band	0.667	0.566	0.745	0.717	0.235
Gamma band	0.841	0.833	0.875	0.868	0.757
All sub-frequency bands	**0.971**	**0.970**	**1.000**	**0.958**	**0.952**
All frequency bands	0.942	0.939	0.980	0.939	0.900

The bold values here is the highest performance among all classifiers.

For the sub-frequency bands, classification performance varied substantially. The alpha and gamma bands showed the strongest discriminative ability, with accuracies of 0.841 and macro-F1 scores of 0.840 and 0.833, respectively. In the alpha band (Figure 6d), the most informative predictors were the explained variance of microstate F and the occurrence of microstates A and C (importance score > 1). In the gamma band (Figure 6f), the top-ranking contributors included the explained variance of microstate E and the occurrence of microstate A (importance scores > 1). The theta band achieved moderate classification performance, with an accuracy of 0.739 and a macro-F1 score of 0.735. In this band, the coverage of microstate D emerged as the most discriminative feature (importance score > 1) (Figure 6c). In contrast, classification performance in the delta and beta bands was relatively poor, with accuracies of 0.623 and 0.667, respectively. The reduced performance in these bands was primarily attributable to difficulties in distinguishing the ROC state from the other two conditions. Specifically, both the delta (Figure 6b) and beta (Figure 6e) band classifiers exhibited low AUC (delta: 0.586, beta: 0.676) and F1 scores (delta: 0.465, beta: 0.235) for the ROC state.

**Figure 7:**
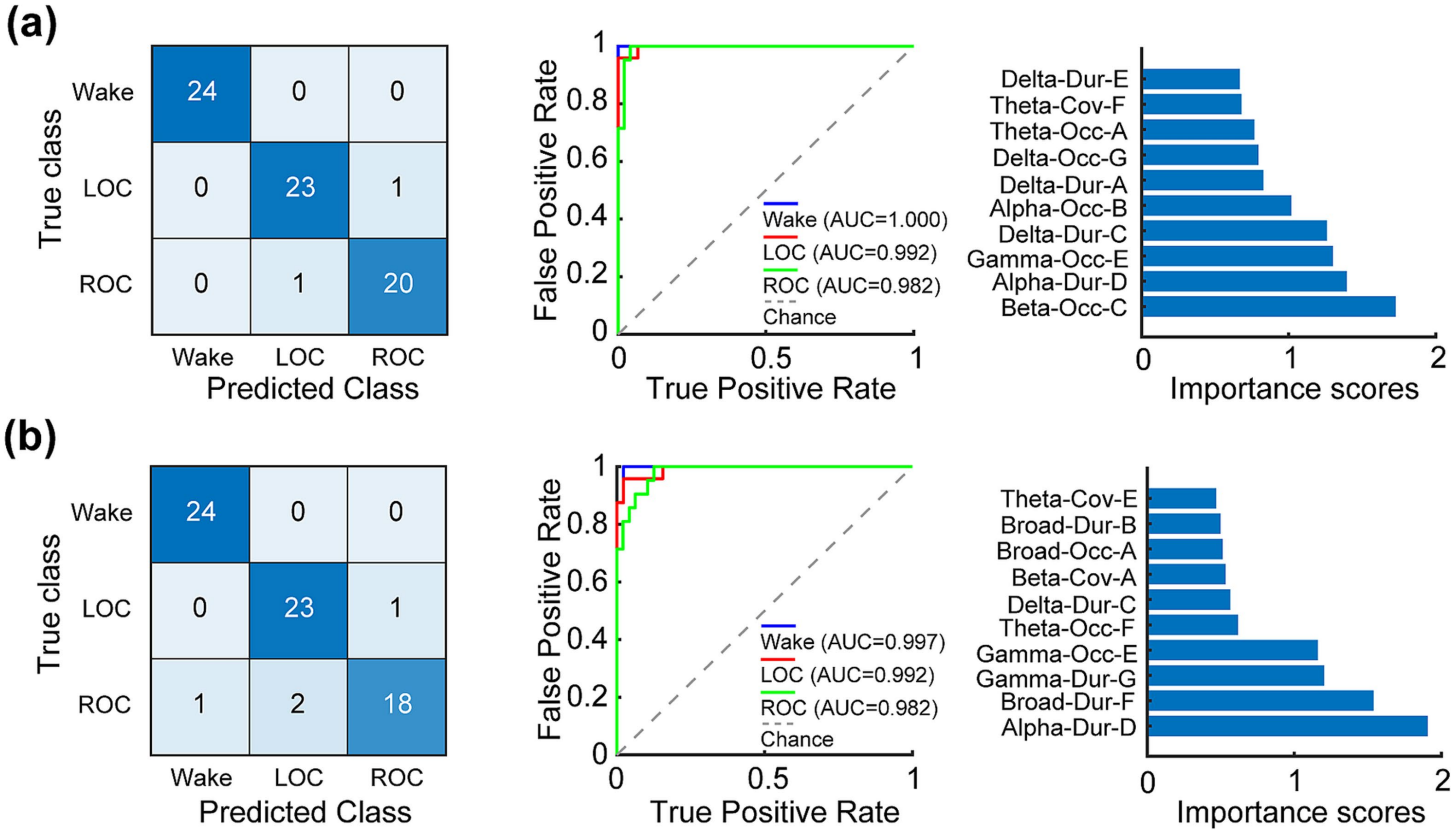
Superior classification of wakefulness, loss of consciousness (LOC), and recovery of consciousness (ROC) achieved by integrating frequency-specific EEG microstate features. Support vector machine (SVM) classifiers were trained on the top-10 microstate features selected via hybrid neighborhood component analysis and minimum redundancy maximum relevance from 63 complete-case samples (21 participants × 3 states). **(a)** Performance using concatenated features from all five sub-frequency bands (delta, theta, alpha, beta, and gamma). **(b)** Performance using features from all six bands (broadband + five sub-bands). Left panels show confusion matrices; middle panels display receiver operating characteristic curves with area under the curve (AUC) values for each state; right panels rank the top-10 features by composite importance score. The high classification accuracy of 0.971 when combining sub-frequency features demonstrates that frequency-specific microstate parameters provide complementary discriminative information masked in conventional broadband analysis and markedly enhance sensitivity for tracking consciousness transitions (see Results 3.4 and Discussion 4.4).

When combining microstate features from all sub-frequency bands (Figure 7a), the classifier achieved the highest performance, surpassing both the broadband and single-band models. The overall accuracy reached 0.971, with a macro-F1 score of 0.970 and per-class F1 scores of 1.00 (wakefulness), 0.958 (LOC), and 0.952 (ROC) (see Table 1). The corresponding AUC values were 1.000, 0.992, and 0.982. Feature-importance analysis indicated that the predictors contributing most strongly to the classification were distributed across multiple frequency bands, including the mean occurrence of microstate C in the beta band, mean duration of microstate D in the alpha band, mean occurrence of microstate E in the gamma band, and mean duration of microstate C in the delta band (importance scores > 1).

A second composite model that incorporated all microstate features across broadband and the six sub-frequency bands (Figure 7b) yielded similarly high performance, with an overall accuracy of 0.942, a macro-F1 score of 0.9398, and per-class F1 scores of 0.980 (wakefulness), 0.939 (LOC), and 0.900 (ROC). The corresponding AUC values were 0.997, 0.992, and 0.982. The features contributing most strongly to classification included the mean duration of microstate D in the alpha band, mean duration of microstate F in the broadband, mean duration of microstate G in the gamma band, and mean occurrence of microstate E in the gamma band (importance scores > 1).

The classification performance of the SVM classifiers using frequency-specific EEG microstate features and five-fold CV is summarized in Table S8. The results showed that SVM performance remained highly stable between CV strategies of LOOCV and five-fold CV (accuracy variation < 2.1% across frequency bands). The classification performance of Random Forest classifiers (see Supplementary Table S9 for LOOCV results and Table S10 for five-fold CV results) yielded comparable, although slightly lower, accuracies (average difference 3.4%) compared with the SVM classifiers. This consistency confirmed that the classification performance was robust and supported the reliability of the frequency-specific microstate features.

## 4 Discussion

By integrating power spectral, microstate, and machine-learning analyses, we characterized how oscillatory and spatiotemporal brain dynamics change during the transition from wakefulness to ciprofol-induced unconsciousness and the subsequent recovery. Ciprofol anesthesia produced distinct frequency-dependent changes in both spectral power and microstate temporal parameters, revealing convergent and complementary markers of anesthetic-induced unconsciousness. Frequency-specific microstates reliably discriminated conscious states with excellent performance. Together, these results delineate the spectral-temporal brain dynamics of ciprofol anesthesia and highlight frequency-specific microstate analysis as a sensitive framework for quantifying anesthetic-induced state transitions.

### 4.1 Spectral signatures of ciprofol-induced unconsciousness

Ciprofol induced pronounced and state-dependent spectral power alterations, characterized by increased delta, theta, and alpha power during LOC, followed by partial normalization during recovery. In contrast, beta and gamma activity remained elevated throughout the ROC period. The topographic distribution of wakefulness-normalized power revealed that low-frequency and alpha enhancements were concentrated over frontal regions, whereas beta and gamma increases were more broadly distributed across the frontal and central cortices. These patterns closely parallel the well-established EEG signatures of propofol anesthesia, which typically include increased slow oscillations and anterior alpha synchronization. These effects are thought to reflect enhanced GABA_A_ receptor-mediated inhibition, cortical up-down state alternations, and disrupted thalamocortical communication (Brown et al., 2011; Ní Mhuircheartaigh et al., 2013; Purdon et al., 2013; Weiner et al., 2023).

The persistent elevation of beta and gamma power during the ROC stage might be related to the gradual reemergence of conscious processing. As reported by Purdon et al., (2013), during propofol anesthesia transitions, the period surrounding LOC and early ROC is characterized by transient high-frequency activity associated with the phenomenon of a “traveling peak” pattern across cortical regions. These beta and gamma oscillations are thought to reflect the gradual dissolution and subsequent reinstatement of corticocortical communication underlying consciousness processing (Zhang et al., 2023). The persistent beta and gamma activity observed with ciprofol may therefore indicate a partial restoration of cortical connectivity and integrative processing preceding the full return of consciousness.

### 4.2 Global explained variance and neural networks of microstates

GEV quantifies the proportion of EEG signal variance accounted for by the identified microstate maps and therefore indicates the stability and representativeness of recurring spatial configurations over time (Michel and Koenig, 2018). Across all frequency bands and consciousness states in the present study, GEV consistently exceeded 70%, demonstrating that the seven extracted microstates robustly captured the dominant spatial patterns of brain activity during ciprofol anesthesia. Moreover, the persistence of multiple identifiable microstate classes during LOC, together with stable explained variance values, suggests that the global topographical organization of functional networks remained structurally preserved even as their dynamic transitions slowed substantially.

Prior source-localization studies have provided a neurophysiological framework for interpreting canonical EEG microstates (Britz et al., 2010; Custo et al., 2017). Specifically, microstate A is associated with the visual network, reflecting occipital processing; microstate B corresponds to the auditory network involving the bilateral superior temporal regions; microstate C is linked to the salience and dorsal attention networks, engaging the fronto-insular and cingulate regions; and microstate D reflects activity within the frontoparietal and executive control networks. In addition to these classical microstates, microstate E has been tentatively associated with default mode network activity, microstate F with limbic and emotion-related circuits, and microstate G with sensorimotor networks. However, the functional interpretations and source localizations of these noncanonical microstates (E–G) remain less well established than those of canonical A–D maps and are still under active investigation.

### 4.3 Temporal reorganization of frequency-specific microstates under ciprofol

Frequency-resolved microstate analysis revealed that ciprofol anesthesia induced a structured and state-dependent reorganization of large-scale cortical dynamics. Across frequency bands, the mean duration, coverage, and occurrence of microstates exhibited systematic changes that paralleled the behavioral transitions from wakefulness to LOC and ROC. These modulations reflect not only the slowing of cortical temporal dynamics but also selective alterations in the functional engagement of distinct resting-state networks represented by specific microstates.

At the broadband level, ciprofol anesthesia produced prolonged microstate durations and reduced occurrence during LOC, followed by recovery during ROC. This pattern indicates a global slowing of cortical state transitions and reduced temporal flexibility, which is consistent with decreased large-scale neural variability during unconsciousness (Tarailis et al., 2024). Similar slowed microstate dynamics have been reported for propofol-induced unconsciousness, sleep, and disorders of consciousness, as reflected by increased mean durations (Brodbeck et al., 2012; Artoni et al., 2022; Toplutaş et al., 2023). Our results suggest that ciprofol disrupts metastable brain states, leading to sparser, longer-lasting microstates under anesthesia. The increased coverage of microstate F aligns with existing reports of noncanonical microstates emerging during propofol sedation, which may reflect stabilized frontal or default mode network-related activity (Shi et al., 2020). Moreover, the reversibility of broadband microstate dynamics during ROC is consistent with the pharmacological profile of ciprofol, a rapidly metabolized GABA_A_ receptor agonist (Li et al., 2025), although direct EEG comparisons remain limited.

In the low-frequency (delta and theta) bands, the most prominent modulation was observed in microstate D, which exhibited reductions in duration, coverage, and occurrence during LOC. Given that microstate D is functionally linked to the frontoparietal and executive control networks (Seitzman et al., 2017), its suppression in low-frequency bands is consistent with impaired top-down executive processing and reduced attentional control during anesthetic-induced unconsciousness (Mashour and Hudetz, 2017). Moreover, in the theta band, the coverage of microstate D contributed most strongly to classification accuracy (73.9%), supporting the role of low-frequency microstate D dynamics in encoding transitions between conscious states.

In the higher-frequency (beta and gamma) bands, microstate alterations were more heterogeneous, with the most consistent state-dependent changes observed in microstates A, C, and E. Microstate A, associated with visual networks, and microstate C, linked to salience and dorsal attention networks, both demonstrated reduced occurrence and coverage during LOC and returned to wakefulness levels during ROC (Tarailis et al., 2024). These reductions likely reflect suppressed sensory processing and attenuated attentional engagement during unconsciousness, followed by reactivation of perceptual and orienting networks as consciousness recovers (Britz et al., 2010). The recovery of these microstates during ROC aligns with evidence that the reemergence of salience and visuospatial attention networks is a key neural signature of returning awareness (Rokos et al., 2021). Microstate E, which has been tentatively associated with executive or self-referential functions, displayed persistent reductions in coverage and occurrence from wakefulness through LOC to ROC, along with significantly decreased explained variance in the gamma band during ROC. This finding suggests lingering gamma-band desynchronization and incomplete restoration of cognitive control networks (Li et al., 2017).

Alpha band microstate features yielded the highest classification accuracy (84.06%) for distinguishing consciousness states and were characterized by the longest durations during wakefulness and the highest occurrence during ROC across multiple microstate classes. Contrary to the longstanding assumption that broadband EEG microstates primarily originate from alpha-band oscillations (Milz et al., 2017), our findings indicate that alpha-microstate dynamics vary markedly across conscious states and diverge from broadband patterns. This observation supports prior studies indicating that broadband microstate dynamics cannot be inferred from isolated frequency bands (Croce et al., 2020; Férat et al., 2022). Furthermore, previous studies have shown that the increased frontal alpha power during propofol sedation originates predominantly from microstate F activity (Shi et al., 2020), which is consistent with our finding of increased alpha-band microstate F coverage during LOC.

### 4.4 Advantages and implications of frequency-specific microstate analysis

This study underscores the methodological and conceptual advantages of frequency-resolved microstate analyses. Neurophysiologically, slow oscillations are often related to large-scale inhibitory synchrony and global state transitions, whereas high-frequency oscillations are linked to localized cortical integration and network communication (Lemieux et al., 2014; Chaudhuri et al., 2018). Conventional broadband microstate analyses can obscure these differential contributions by collapsing across distinct oscillatory mechanisms. Supporting this view, previous studies have shown that alpha-band microstate parameters outperform broadband-derived metrics in predicting both eye-open and eye-closed states and anxiety levels (Férat et al., 2022; Xue et al., 2025), reinforcing the value of spectrally specific microstate analyses for uncovering novel neural markers in both basic and clinical neuroscience.

By independently characterizing microstates across canonical frequency bands, this study revealed unique frequency-dependent reorganization patterns associated with transitions between consciousness states. The comparatively low classification accuracy (<0.70) observed for the delta and beta bands may be attributed to the inherent characteristics of these bands under anesthesia. In the delta band, a pronounced increase in slow-wave activity during LOC produces highly stereotyped neural activity, prolonged microstate durations, and reduced temporal variability (Zhang et al., 2025). Our results showed that these trends did not recover immediately during the ROC analysis, which may limit the discriminative power of microstate features for distinguishing between LOC and ROC. The beta band is often associated with residual muscle artifacts and less stable topographic patterns (Muthukumaraswamy, 2013), which may lead to poorer feature separability when discriminating between wakefulness and ROC states, as reflected by the low F1-ROC score (0.235).

By contrast, the alpha and gamma bands yielded an accuracy of 0.841, highlighting their greater sensitivity to consciousness-related network dynamics. Alpha oscillations are closely associated with anteriorization and thalamocortical disruption (Purdon et al., 2013; Vijayan et al., 2013), whereas gamma oscillations are sensitive to high-frequency binding and network desynchronization, which are critical for conscious processing (Sleigh et al., 2001; Li et al., 2017). These findings align with those of prior propofol studies demonstrating superior state discrimination using alpha brain networks and spectral slopes of high-frequency bands (Chennu et al., 2016; Weyer et al., 2021; Zhang et al., 2023, 2025).

Overall, classification models that integrated features from all sub-frequency bands achieved superior accuracy compared with broadband models, indicating that the microstate features from different bands carried complementary rather than redundant information about brain-state dynamics. Although conventional broadband microstate analysis provides a useful global description of cortical activity, frequency-resolved features allow the identification of band-specific contributions to neural network organization. Thus, the frequency-resolved microstate framework provides a mechanistically grounded bridge between oscillatory rhythms and transient functional networks, offering a refined analytical lens for investigating the neural correlates of consciousness and enhancing the precision of anesthetic depth monitoring.

Given the exploratory nature of this study, no *a priori* sample-size calculations were performed. Nevertheless, the sample size was consistent with those used in prior EEG microstate studies in anesthetic contexts, which typically included 10–23 participants (Zhang et al., 2021; Artoni et al., 2022; Lapointe et al., 2023; Liang et al., 2024). A post-hoc power analysis (G*Power, repeated-measures ANOVA, effect size *f* = 0.3 (medium effect), significance level = 0.05, correlation among repeated measures = 0.5, nonsphericity correction = 1) confirmed that the study provided approximately 74% statistical power for the main effects observed.

The present study was limited to young, healthy male participants. This homogeneity minimized inter-individual variability but restricted generalizability to females and older adults. Moreover, prior studies have reported subtle influences of sex on EEG alpha power and microstate dynamics (Hill et al., 2023), as well as age-related changes in alpha band activity and obvious age effects on the mean occurrence and duration of microstates during anesthesia (Purdon et al., 2015a; Lapointe et al., 2023). Therefore, the frequency-specific microstate patterns identified here may differ in magnitude or topography in female and older populations. Future studies should include mixed-sex and broader age range cohorts to evaluate the robustness and clinical translatability of these markers.

## 5 Conclusion

In summary, this study comprehensively analyzed frequency-specific EEG microstate correlates of ciprofol-induced alterations in consciousness. Ciprofol anesthesia induced canonical low-frequency power increases and alpha modulation similar to propofol but preserved higher-frequency (beta and gamma) activity. Frequency-resolved microstate analysis revealed distinct frequency band-dependent patterns of neural reconfiguration that, when combined, enabled a highly accurate classification of consciousness states. These findings deepen our understanding of how ciprofol modulates large-scale cortical dynamics and highlight the value of frequency-specific EEG microstate features as potential quantitative biomarkers for monitoring anesthetic depth and neural state transitions.

## Data Availability

The raw data supporting the conclusions of this article will be made available by the authors, without undue reservation.
