# Supplementary material for: Frequency-specific microstate correlates of ciprofol-induced alterations of consciousness
Source: Front Neurosci. 2026 Apr 7;20:1781321. doi: 10.3389/fnins.2026.1781321 (PMC13096086; doi:10.3389/fnins.2026.1781321)
Supplement: Supplementary file 1 [file Data_Sheet_1.docx]

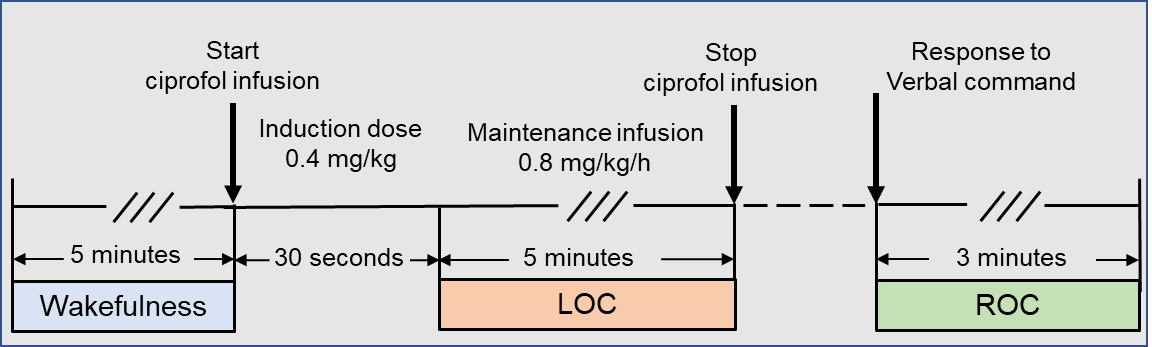


**Figure S1. The ciprofol administration scheme.** Before ciprofol administration, participants were instructed to remain relaxed and awake for 5 minutes to establish baseline recordings. An induction dose of ciprofol at 0.4mg/kg was then administered intravenously over 30 seconds, followed by a maintenance infusion of 0.8 mg/kg/h for 5 minutes. The infusion was subsequently discontinued. Participants were considered awake upon their first correct response to the verbal command “open your eyes.” After recording the EEG data for another 3 minutes, the experiment was stopped. The EEG segments recorded during baseline were used in analysis for wakefulness (5 minutes). The EEG recorded during maintenance infusion stage were used in analysis for loss of consciousness (LOC, 5 minutes). The EEG segments recorded after behavioral recovery were used in analysis for recovery of consciousness (ROC, 3 minutes).


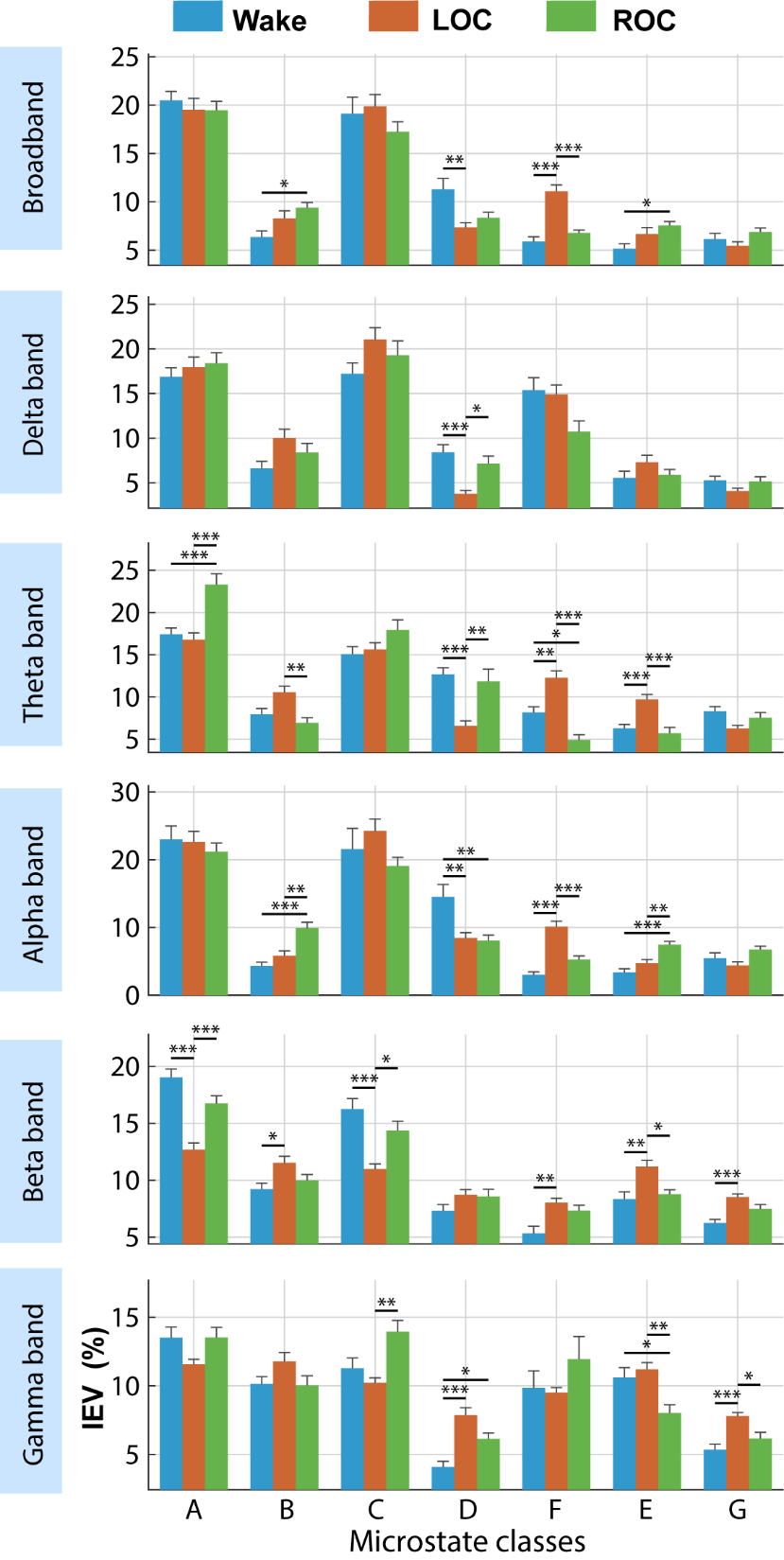


**Figure S2. Frequency-specific individual explained variance (IEV) of microstates across consciousness states under ciprofol anesthesia.** Group-level comparisons of IEV for seven canonical microstates (A–G) across three consciousness states—wakefulness (WAKE), loss of consciousness (LOC), and recovery of consciousness (ROC)—for the broadband (1-45 Hz), delta (1–4 Hz), theta (4–8 Hz), alpha (8–13 Hz), beta (13–30 Hz), and gamma (30–45 Hz) bands. The error bars represent mean ± SEM. One-way repeated measure ANOVA was applied across states, followed by Bonferroni-corrected pairwise comparisons. *P < 0.05, **P < 0.01, ***P < 0.001.

**Table S1.** Statistical comparisons of **individual explained variance** of microstates from each frequency band across wakefulness, loss of consciousness (LOC), and return of consciousness (ROC) states under ciprofol anesthesia.

| **Frequency bands** | **Wake** | **LOC** | **ROC** | **F value** | **P value** |
| --- | --- | --- | --- | --- | --- |
| **Broadband** | | | | | |
| A | 19.58(12.52, 27.99) | 17.98(9.07, 29.37) | 19.27(14.14, 32.72) | 0.50 | 0.563 |
| B | 6.37(1.77, 13.23) | 7.90(3.94, 21.38) | 9.45(5.15, 17.04) | 8.43 | **0.004** |
| C | 16.37(11.47, 45.73) | 20.56(8.43, 29.22) | 16.06(11.71, 30.44) | 1.21 | 0.303 |
| D | 10.39(5.36, 27.40) | 7.48(2.78, 12.61) | 7.82(2.46, 14.94) | 9.15 | **0.004** |
| F | 5.65(1.94, 12.17) | 10.87(5.82, 18.38) | 6.37(4.62, 9.33) | 38.82 | **<0.001** |
| E | 5.38(0.93, 8.95) | 5.67(3.25, 17.30) | 7.45(3.72, 11.48) | 6.47 | **0.007** |
| G | 6.76(1.34, 11.02) | 4.73(3.42, 9.38) | 7.33(2.66, 9.96) | 3.72 | **0.038** |
| **Delta band** | | | | | |
| A | 16.28(10.03, 30.43) | 17.95(7.54, 28.47) | 17.29(11.21, 32.97) | 0.69 | 0.501 |
| B | 5.57(0.36, 17.10) | 9.18(4.43, 24.16) | 7.61(1.62, 19.86) | 4.66 | **0.018** |
| C | 14.83(9.88, 31.34) | 21.90(7.62, 29.88) | 19.43(8.20, 42.78) | 2.77 | 0.098 |
| D | 8.19(2.09, 16.37) | 3.69(1.37, 8.79) | 6.91(0.75, 17.23) | 11.71 | **0.001** |
| F | 13.68(6.97, 31.06) | 13.99(7.36, 28.98) | 8.78(5.71, 24.99) | 3.82 | **0.032** |
| E | 4.63(0.54, 12.64) | 6.42(3.42, 18.29) | 5.26(1.92, 11.48) | 2.03 | 0.145 |
| G | 5.03(0.54, 9.99) | 3.69(2.09, 7.56) | 5.34(0.71, 10.57) | 2.49 | 0.113 |
| **Theta band** | | | | | |
| A | 18.16(8.92, 22.15) | 16.21(8.38, 25.33) | 22.60(15.57, 37.05) | 13.53 | **<0.001** |
| B | 8.51(3.34, 16.85) | 10.04(7.32, 21.57) | 6.02(3.28, 12.88) | 11.13 | **<0.001** |
| C | 14.60(8.28, 23.76) | 15.03(8.56, 25.53) | 16.95(10.64, 28.68) | 3.66 | 0.056 |
| D | 11.61(6.37, 20.51) | 6.08(2.65, 15.31) | 11.62(1.17, 27.11) | 15.49 | **<0.001** |
| F | 7.82(2.84, 14.60) | 12.21(5.65, 21.75) | 4.08(1.14, 13.05) | 33.49 | **<0.001** |
| E | 6.52(1.96, 10.94) | 10.08(5.06, 15.15) | 5.24(2.05, 15.20) | 20.21 | **<0.001** |
| G | 8.57(4.08, 12.17) | 5.99(4.24, 10.30) | 7.70(1.79, 12.30) | 7.21 | **0.003** |
| **Alpha band** | | | | | |
| A | 22.90(6.81, 42.52) | 19.61(11.94, 37.65) | 20.71(14.56, 40.25) | 0.47 | 0.546 |
| B | 3.95(0.44, 8.80) | 5.81(1.65, 18.00) | 9.37(3.78, 19.60) | 26.20 | **<0.001** |
| C | 19.19(7.05, 69.41) | 24.39(13.42, 41.40) | 17.79(8.61, 31.36) | 1.58 | 0.224 |
| D | 10.85(5.40, 35.88) | 7.74(1.55, 15.02) | 6.53(1.21, 14.95) | 10.33 | **0.003** |
| F | 2.70(0.86, 7.15) | 10.72(4.15, 17.04) | 4.69(1.66, 10.67) | 47.55 | **<0.001** |
| E | 2.70(0.13, 8.35) | 4.35(1.48, 10.19) | 7.46(3.32, 11.13) | 16.51 | **<0.001** |
| G | 5.17(0.14, 12.88) | 3.70(1.50, 9.70) | 7.21(1.80, 11.64) | 4.76 | **0.022** |
| **Table S1. (Continued)** | | | | | |
| **Frequency bands** | **Wake** | **LOC** | **ROC** | **F value** | **P value** |
| **Beta band** | | | | | |
| A | 19.19(12.06, 27.48) | 12.49(7.98,17.29) | 16.46(11.94, 22.14) | 39.74 | **<0.001** |
| B | 9.40(5.75, 15.31) | 10.86(7.35, 17.89) | 9.45(5.33, 16.66) | 11.83 | **<0.001** |
| C | 15.23(10.47,28.21) | 10.99(6.95, 14.61) | 13.85(8.29,24.18) | 14.79 | **<0.001** |
| D | 6.90(2.33, 12.19) | 8.72(5.30, 12.93) | 8.21(3.51, 14.54) | 2.95 | 0.074 |
| F | 4.63(2.16, 13.26) | 7.95(4.97, 13.69) | 7.11(3.86, 12.65) | 12.00 | **0.001** |
| E | 7.94(3.66, 16.59) | 11.05(6.17, 16.91) | 8.53(6.06, 12.73) | 11.32 | **<0.001** |
| G | 6.79(2.99, 8.19) | 8.37(6.06, 10.70) | 7.54(3.61, 10.28) | 23.15 | **<0.001** |
| **Gamma band** | | | | | |
| A | 12.85(6.33, 22.16) | 11.41(9.04, 15.25) | 13.81(4.56, 19.19) | 2.92 | 0.076 |
| B | 9.34(6.71, 14.62) | 11.92(7.79, 20.19) | 10.15(1.42, 19.23) | 3.36 | 0.056 |
| C | 10.41(6.57, 18.67) | 10.13(7.26, 14.86) | 12.59(9.75, 21.63) | 10.91 | **<0.001** |
| D | 4.36(0.82, 8.81) | 7.66(3.95, 14.42) | 6.55(0.70, 8.74) | 22.68 | **<0.001** |
| F | 8.42(3.34, 26.91) | 9.13(6.89, 13.60) | 10.56(4.48, 41.34) | 1.16 | 0.312 |
| E | 9.97(6.08, 18.12) | 11.17(6.16, 15.12) | 7.51(1.05, 15.03) | 9.37 | **0.001** |
| G | 5.01(2.37, 8.67) | 7.65(5.85, 10.46) | 6.43(0.41, 8.82) | 14.48 | **<0.001** |
| *Note: Data are expressed as median (minimum, maximum). One-way repeated measure ANOVA was used to compare differences across conscious states under ciprofol-induced anesthesia. Indicators A–G correspond to seven microstates (detailed in Methods). P < 0.05 indicates significant group differences.* | | | | | |

**Table S2.** Statistical comparisons of EEG microstate features from **the broad frequency band** across wakefulness, loss of consciousness (LOC), and return of consciousness (ROC) states under ciprofol anesthesia.

| **Features** | **Wake** | **LOC** | **ROC** | **F value** | **P value** |
| --- | --- | --- | --- | --- | --- |
| **meanDuration (s)** | | | | | |
| A | 0.046(0.033, 0.067) | 0.054(0.038, 0.064) | 0.039(0.031, 0.059) | 30.65 | **< 0.001** |
| B | 0.033(0.028, 0.047) | 0.045(0.034, 0.062) | 0.033(0.028, 0.039) | 64.03 | **< 0.001** |
| C | 0.038(0.030, 0.074) | 0.053(0.037, 0.069) | 0.035(0.030, 0.048) | 26.18 | **< 0.001** |
| D | 0.036(0.025, 0.063) | 0.044(0.036, 0.050) | 0.031(0.022, 0.038) | 23.90 | **< 0.001** |
| F | 0.031(0.027, 0.043) | 0.047(0.033, 0.056) | 0.031(0.024, 0.039) | 117.91 | **< 0.001** |
| E | 0.029(0.025, 0.041) | 0.041(0.029, 0.054) | 0.030(0.024, 0.039) | 75.42 | **< 0.001** |
| G | 0.031(0.024, 0.046) | 0.039(0.031, 0.044) | 0.028(0.023, 0.034) | 53.92 | **< 0.001** |
| **Coverage (%)** | | | | | |
| A | 21.47(15.64, 28.38) | 18.91(11.80, 29.21) | 21.16(15.98, 30.49) | 1.09 | 0.335 |
| B | 11.72(5.00, 19.12) | 12.22(7.57, 25.67) | 15.15(10.29, 22.71) | 8.59 | **0.003** |
| C | 16.87(13.10, 42.17) | 19.72(10.53, 26.15) | 16.48(12.89, 26.81) | 1.32 | 0.275 |
| D | 15.33(9.33, 28.76) | 11.94(6.19, 18.25) | 12.34(5.66, 20.52) | 9.99 | **0.003** |
| F | 10.72(6.66, 18.09) | 15.10(10.53, 22.21) | 10.75(8.99, 14.63) | 26.34 | **< 0.001** |
| E | 10.63(3.48, 13.85) | 9.63(6.41, 20.19) | 12.06(7.12, 15.54) | 6.02 | **0.008** |
| G | 11.33(3.72, 16.19) | 8.73(6.53, 13.63) | 11.05(5.95, 13.69) | 3.48 | 0.058 |
| **MeanOccurrence (/s)** | | | | | |
| A | 5.224(3.638, 6.399) | 3.979(2.375, 4.917) | 5.375(4.333, 7.012) | 43.16 | **< 0.001** |
| B | 3.642(1.312, 5.819) | 2.668(1.808, 4.168) | 4.533(2.656, 6.398) | 28.53 | **< 0.001** |
| C | 4.478(3.355, 5.804) | 3.705(2.253, 4.486) | 4.695(3.860, 7.429) | 21.31 | **< 0.001** |
| D | 4.085(2.978, 5.454) | 2.644(1.671, 4.981) | 4.023(2.471, 6.524) | 30.15 | **< 0.001** |
| F | 3.381(1.932, 5.861) | 3.315(2.361, 4.025) | 3.687(2.723, 4.843) | 3.50 | 0.062 |
| E | 3.301(1.082, 5.276) | 2.348(1.548, 3.774) | 4.017(2.409, 5.349) | 21.92 | **< 0.001** |
| G | 3.721(1.131, 4.992) | 2.205(1.685, 3.947) | 3.747(1.988, 4.873) | 22.27 | **< 0.001** |
| *Note: Data are expressed as median (minimum, maximum). One-way repeated measure ANOVA was used to compare differences across conscious states under ciprofol-induced anesthesia. Indicators A–G correspond to seven microstates (detailed in Methods). P < 0.05 indicates significant group differences.* | | | | | |

**Table S3.** Statistical comparisons of EEG microstate features from **the delta (1-4 Hz) frequency band** across wake, loss of consciousness (LOC), and return of consciousness (ROC) states under ciprofol anesthesia.

| **Features** | **Wake** | **LOC** | **ROC** | **F value** | **P value** |
| --- | --- | --- | --- | --- | --- |
| **meanDuration (s)** | | | | | |
| A | 0.287(0.255, 0.391) | 0.327(0.298, 0.407) | 0.304(0.260, 0.449) | 12.53 | **< 0.001** |
| B | 0.242(0.211, 0.315) | 0.313(0.258, 0.431) | 0.272(0.221, 0.341) | 23.39 | **< 0.001** |
| C | 0.279(0.259, 0.344) | 0.335(0.295, 0.412) | 0.314(0.250, 0.411) | 14.04 | **< 0.001** |
| D | 0.258(0.224, 0.313) | 0.259(0.228, 0.312) | 0.283(0.229, 0.318) | 4.09 | **0.027** |
| F | 0.298(0.259, 0.440) | 0.338(0.271, 0.384) | 0.274(0.237, 0.420) | 4.08 | **0.027** |
| E | 0.233(0.204, 0.313) | 0.280(0.243, 0.388) | 0.257(0.229, 0.303) | 13.98 | **< 0.001** |
| G | 0.235(0.194, 0.263) | 0.257(0.223, 0.283) | 0.255(0.216, 0.313) | 9.15 | **0.001** |
| **Coverage (%)** | | | | | |
| A | 17.49(12.68, 28.95) | 18.89(9.53, 27.15) | 18.99(14.14, 30.06) | 1.55 | 0.225 |
| B | 10.31(2.32, 20.34) | 14.42(8.61, 28.56) | 13.19(5.73, 23.24) | 7.35 | **0.004** |
| C | 15.07(12.43, 27.40) | 19.93(9.34, 26.78) | 18.45(10.95, 35.63) | 2.00 | 0.165 |
| D | 13.38(5.86, 20.66) | 7.65(3.86, 16.52) | 12.90(4.35, 22.40) | 13.77 | **< 0.001** |
| F | 19.85(11.53, 39.25) | 19.17(13.12, 29.44) | 13.29(9.09, 28.01) | 7.27 | **0.004** |
| E | 8.67(2.97, 18.81) | 11.19(6.76, 21.98) | 10.48(6.33, 15.38) | 2.22 | 0.126 |
| G | 9.27(3.25, 14.86) | 8.25(5.61, 12.55) | 10.67(3.96, 15.05) | 4.15 | **0.031** |
| **meanOccurrence (/s)** | | | | | |
| A | 0.610(0.439, 0.755) | 0.586(0.301, 0.738) | 0.618(0.498, 0.800) | 2.78 | 0.075 |
| B | 0.434(0.109, 0.646) | 0.474(0.298, 0.664) | 0.476(0.212, 0.767) | 1.47 | 0.243 |
| C | 0.555(0.427, 0.810) | 0.609(0.310, 0.741) | 0.600(0.389, 0.869) | 0.31 | 0.662 |
| D | 0.529(0.249, 0.772) | 0.301(0.124, 0.596) | 0.443(0.187, 0.736) | 19.60 | **< 0.001** |
| F | 0.660(0.427, 0.895) | 0.565(0.440, 0.772) | 0.494(0.351, 0.669) | 20.91 | **< 0.001** |
| E | 0.369(0.134, 0.682) | 0.409(0.278, 0.566) | 0.403(0.272, 0.541) | 0.18 | 0.784 |
| G | 0.410(0.166, 0.605) | 0.327(0.223, 0.444) | 0.419(0.171, 0.523) | 7.03 | **0.006** |
| *Note: Data are expressed as median (minimum, maximum). One-way repeated measure ANOVA was used to compare differences across conscious states under ciprofol-induced anesthesia. Indicators A–G correspond to seven microstates (detailed in Methods). P < 0.05 indicates significant group differences.* | | | | | |

**Table S4.** Statistical comparisons of EEG microstate features from **the theta (4-8 Hz) frequency band** across wake, loss of consciousness (LOC), and return of consciousness (ROC) states under ciprofol anesthesia.

| **Features** | **Wake** | **LOC** | **ROC** | **F value** | **P value** |
| --- | --- | --- | --- | --- | --- |
| **meanDuration (s)** | | | | | |
| A | 0.154(0.129, 0.175) | 0.154(0.136, 0.171) | 0.162(0.135, 0.275) | 5.30 | **0.026** |
| B | 0.140(0.117, 0.178) | 0.145(0.124, 0.181) | 0.141(0.113, 0.179) | 1.65 | 0.211 |
| C | 0.140(0.129, 0.185) | 0.142(0.123, 0.167) | 0.148(0.118, 0.210) | 3.12 | 0.076 |
| D | 0.152(0.143, 0.180) | 0.137(0.114, 0.169) | 0.143(0.110, 0.256) | 7.32 | **0.007** |
| F | 0.146(0.116, 0.183) | 0.157(0.131, 0.191) | 0.131(0.112, 0.158) | 13.47 | **< 0.001** |
| E | 0.134(0.106, 0.162) | 0.145(0.128, 0.163) | 0.130(0.115, 0.179) | 6.75 | **0.003** |
| G | 0.141(0.109, 0.161) | 0.134(0.117, 0.147) | 0.139(0.102, 0.209) | 2.40 | 0.119 |
| **Coverage (%)** | | | | | |
| A | 18.06(11.78, 21.10) | 17.42(10.90, 22.30) | 22.31(16.33, 33.88) | 16.95 | **< 0.001** |
| B | 13.19(7.33, 21.51) | 14.42(11.53, 24.80) | 11.67(7.98, 19.15) | 8.86 | **0.001** |
| C | 14.00(9.67, 22.05) | 15.06(10.50, 21.56) | 16.69(10.73, 24.83) | 6.00 | **0.012** |
| D | 16.56(11.26, 22.63) | 10.52(6.44, 19.76) | 16.57(3.99, 29.48) | 18.85 | **< 0.001** |
| F | 13.05(8.18, 19.47) | 16.85(10.36, 25.95) | 8.66(4.78, 16.50) | 37.24 | **< 0.001** |
| E | 10.99(6.58, 16.40) | 14.19(9.11, 18.49) | 10.21(6.66, 19.08) | 18.93 | **< 0.001** |
| G | 13.15(8.32, 16.72) | 10.53(8.38, 15.08) | 12.49(4.86, 18.25) | 7.88 | **0.002** |
| **meanOccurrence (/s)** | | | | | |
| A | 1.173(0.867, 1.500) | 1.107(0.797, 1.432) | 1.286(1.114, 1.601) | 15.95 | **< 0.001** |
| B | 0.923(0.609, 1.212) | 1.018(0.857, 1.379) | 0.881(0.469, 1.238) | 9.76 | **0.001** |
| C | 1.019(0.747, 1.575) | 1.046(0.846, 1.296) | 1.115(0.762, 1.450) | 3.89 | 0.131 |
| D | 1.057(0.778, 1.546) | 0.794(0.531, 1.176) | 1.059(0.361, 1.447) | 17.24 | **< 0.001** |
| F | 0.886(0.670, 1.081) | 1.049(0.789, 1.365) | 0.647(0.377, 1.104) | 46.48 | **< 0.001** |
| E | 0.822(0.507, 1.014) | 0.971(0.710, 1.176) | 0.773(0.498, 1.141) | 17.29 | **< 0.001** |
| G | 0.922(0.631, 1.141) | 0.796(0.689, 1.037) | 0.882(0.416, 1.197) | 5.78 | **0.011** |
| *Note: Data are expressed as median (minimum, maximum). One-way repeated measure ANOVA was used to compare differences across conscious states under ciprofol-induced anesthesia. Indicators A–G correspond to seven microstates (detailed in Methods). P < 0.05 indicates significant group differences.* | | | | | |

**Table S5.** Statistical comparisons of EEG microstate features from the **alpha (8-15 Hz) frequency band** across wake, loss of consciousness (LOC), and return of consciousness (ROC) states under ciprofol anesthesia.

| **Features** | **Wake** | **LOC** | **ROC** | **F value** | **P value** |
| --- | --- | --- | --- | --- | --- |
| **meanDuration (s)** | | | | | |
| A | 0.131(0.099, 0.222) | 0.119(0.090, 0.165) | 0.098(0.078, 0.141) | 20.20 | **< 0.001** |
| B | 0.095(0.084, 0.123) | 0.094(0.073, 0.104) | 0.090(0.074, 0.107) | 6.69 | **0.007** |
| C | 0.124(0.083, 0.450) | 0.129(0.082, 0.162) | 0.087(0.072, 0.113) | 6.53 | **0.015** |
| D | 0.135(0.093, 0.250) | 0.103(0.082, 0.119) | 0.082(0.063, 0.115) | 33.41 | **< 0.001** |
| F | 0.099(0.075, 0.135) | 0.113(0.086, 0.138) | 0.077(0.059, 0.097) | 27.50 | **< 0.001** |
| E | 0.098(0.075, 0.119) | 0.087(0.065, 0.113) | 0.082(0.071, 0.120) | 5.71 | **0.008** |
| G | 0.101(0.069, 0.153) | 0.083(0.071, 0.104) | 0.076(0.062, 0.094) | 25.46 | **< 0.001** |
| **Coverage (%)** | | | | | |
| A | 23.87(9.32, 37.42) | 20.35(14.19, 37.13) | 20.10(14.39, 33.88) | 1.71 | 0.203 |
| B | 10.44(2.71, 14.98) | 9.80(4.26, 20.63) | 16.05(8.81, 21.72) | 36.57 | **< 0.001** |
| C | 17.27(9.25, 69.39) | 23.38(13.62, 35.01) | 15.75(9.71, 25.11) | 2.42 | 0.125 |
| D | 18.62(10.30, 39.79) | 12.66(3.89, 19.54) | 11.74(3.88, 22.45) | 14.19 | **0.001** |
| F | 6.60(3.56, 11.67) | 15.78(8.00, 21.71) | 9.14(4.08, 16.21) | 40.12 | **< 0.001** |
| E | 7.57(0.77, 13.60) | 7.37(3.79, 13.11) | 12.79(7.92, 20.73) | 17.06 | **< 0.001** |
| G | 11.23(0.74, 21.13) | 7.80(3.91, 14.35) | 11.45(5.43, 15.13) | 6.32 | **0.009** |
| **meanOccurrence (/s)** | | | | | |
| A | 1.678(0.755, 2.188) | 1.787(1.526, 2.283) | 2.028(1.681, 2.495) | 12.93 | **< 0.001** |
| B | 1.027(0.291, 1.628) | 1.030(0.521, 1.990) | 1.845(1.134, 2.321) | 50.61 | **< 0.001** |
| C | 1.559(0.966, 1.812) | 1.825(1.493, 2.221) | 1.900(1.161, 2.304) | 20.53 | **< 0.001** |
| D | 1.396(0.823, 1.800) | 1.228(0.468, 1.828) | 1.435(0.607, 2.222) | 2.31 | 0.130 |
| F | 0.813(0.298, 1.395) | 1.387(0.893, 1.640) | 1.141(0.549, 1.778) | 33.29 | **< 0.001** |
| E | 0.723(0.094, 1.489) | 0.841(0.520, 1.508) | 1.609(1.027, 2.039) | 30.55 | **< 0.001** |
| G | 1.052(0.104, 1.634) | 0.897(0.506, 1.581) | 1.457(0.865, 1.906) | 16.31 | **< 0.001** |
| *Note: Data are expressed as median (minimum, maximum). One-way repeated measure ANOVA was used to compare differences across conscious states under ciprofol-induced anesthesia. Indicators A–G correspond to seven microstates (detailed in Methods). P < 0.05 indicates significant group differences.* | | | | | |

**Table S6.** Statistical comparisons of EEG microstate features from the **beta (15-30 Hz) frequency band** across wake, loss of consciousness (LOC), and return of consciousness (ROC) states under ciprofol anesthesia.

| **Features** | **Wake** | **LOC** | **ROC** | **F value** | **P value** |
| --- | --- | --- | --- | --- | --- |
| **meanDuration (s)** | | | | | |
| A | 0.049(0.039, 0.063) | 0.045(0.037, 0.052) | 0.049(0.040, 0.054) | 7.22 | **0.008** |
| B | 0.042(0.036, 0.054) | 0.046(0.043, 0.051) | 0.045(0.036, 0.052) | 6.77 | **0.003** |
| C | 0.042(0.034, 0.064) | 0.040(0.034, 0.043) | 0.042(0.036, 0.047) | 4.14 | **0.048** |
| D | 0.042(0.030, 0.050) | 0.045(0.038, 0.052) | 0.043(0.034, 0.052) | 3.91 | 0.051 |
| F | 0.035(0.031, 0.050) | 0.043(0.039, 0.051) | 0.041(0.033, 0.050) | 16.53 | **< 0.001** |
| E | 0.041(0.034, 0.046) | 0.044(0.039, 0.049) | 0.042(0.035, 0.047) | 16.98 | **< 0.001** |
| G | 0.036(0.029, 0.045) | 0.042(0.037, 0.045) | 0.039(0.032, 0.046) | 14.62 | **< 0.001** |
| **Coverage (%)** | | | | | |
| A | 20.92(14.80, 27.85) | 15.25(10.98, 19.06) | 18.28(14.47, 23.33) | 41.17 | **< 0.001** |
| B | 15.49(12.04, 22.99) | 16.64(12.53, 22.29) | 15.17(10.52, 22.14) | 3.20 | 0.059 |
| C | 16.41(12.18, 25.26) | 12.09(8.40, 14.89) | 13.96(9.59, 21.86) | 21.44 | **< 0.001** |
| D | 12.04(5.72, 16.77) | 14.00(9.74, 18.37) | 13.56(6.98, 19.66) | 4.95 | **0.015** |
| F | 9.34(5.74, 18.25) | 12.50(9.69, 19.16) | 11.78(7.67, 17.37) | 13.13 | **0.001** |
| E | 13.67(9.02, 17.99) | 15.84(10.63, 20.58) | 13.15(10.26, 17.54) | 10.91 | **< 0.001** |
| G | 11.64(6.94, 12.84) | 12.88(9.97, 14.91) | 11.87(7.34, 14.87) | 15.83 | **< 0.001** |
| **meanOccurrence (/s)** | | | | | |
| A | 4.356(3.154, 5.286) | 3.428(2.654, 3.930) | 3.908(3.074, 4.755) | 47.33 | **< 0.001** |
| B | 3.544(2.746, 4.697) | 3.542(2.912, 4.577) | 3.412(2.918, 4.333) | 1.00 | 0.356 |
| C | 3.831(3.360, 4.814) | 2.989(2.234, 3.518) | 3.397(2.578, 4.701) | 34.96 | **< 0.001** |
| D | 2.787(1.851, 3.607) | 3.151(2.489, 3.657) | 3.096(2.015, 4.334) | 3.65 | **0.037** |
| F | 2.416(1.597, 4.345) | 2.965(2.479, 3.795) | 2.843(2.093, 4.102) | 6.16 | **0.016** |
| E | 3.268(2.251, 4.440) | 3.620(2.705, 4.281) | 3.182(2.752, 3.847) | 3.52 | 0.062 |
| G | 2.930(1.859, 3.739) | 3.148(2.633, 3.487) | 3.019(2.205, 3.535) | 2.52 | 0.112 |
| *Note: Data are expressed as median (minimum, maximum). One-way repeated measure ANOVA was used to compare differences across conscious states under ciprofol-induced anesthesia. Indicators A–G correspond to seven microstates (detailed in Methods). P < 0.05 indicates significant group differences.* | | | | | |

**Table S7.** Statistical comparisons of EEG microstate features from the **gamma (30-45 Hz) frequency band** across wake, loss of consciousness (LOC), and return of consciousness (ROC) states under ciprofol anesthesia.

| **Features** | **Wake** | **LOC** | **ROC** | **F value** | **P value** |
| --- | --- | --- | --- | --- | --- |
| **meanDuration (s)** | | | | | |
| A | 0.032(0.027, 0.038) | 0.031(0.028, 0.035) | 0.032(0.029, 0.036) | 0.39 | 0.643 |
| B | 0.033(0.029, 0.038) | 0.036(0.031, 0.041) | 0.033(0.025, 0.041) | 9.42 | **0.001** |
| C | 0.026(0.024, 0.032) | 0.027(0.025, 0.029) | 0.029(0.025, 0.035) | 6.37 | **0.005** |
| D | 0.026(0.018, 0.032) | 0.032(0.026, 0.037) | 0.030(0.024, 0.036) | 33.59 | **< 0.001** |
| F | 0.029(0.024, 0.046) | 0.034(0.029, 0.037) | 0.034(0.026, 0.056) | 3.06 | 0.072 |
| E | 0.031(0.027, 0.037) | 0.034(0.029, 0.037) | 0.030(0.024, 0.034) | 14.13 | **< 0.001** |
| G | 0.025(0.021, 0.028) | 0.029(0.026, 0.033) | 0.028(0.022, 0.033) | 26.24 | **< 0.001** |
| **Coverage (%)** | | | | | |
| A | 17.83(12.08, 25.69) | 14.15(12.67, 17.62) | 16.20(11.33, 21.93) | 8.43 | **0.002** |
| B | 16.54(13.49, 24.63) | 17.84(12.67, 24.04) | 16.10(7.94, 25.14) | 2.36 | 0.120 |
| C | 13.32(9.64, 20.34) | 11.38(9.32, 13.43) | 13.49(10.94, 22.96) | 9.68 | **0.001** |
| D | 9.14(2.84, 14.56) | 12.56(8.01, 19.93) | 12.07(6.06, 14.31) | 15.70 | **< 0.001** |
| F | 14.00(8.03, 27.71) | 14.82(11.85, 19.12) | 16.04(8.76, 40.17) | 1.21 | 0.301 |
| E | 16.36(12.11, 23.13) | 16.61(11.27, 19.88) | 13.19(7.23, 19.67) | 12.49 | **< 0.001** |
| G | 9.99(6.13, 14.04) | 12.02(10.50, 14.91) | 10.89(3.60, 13.64) | 8.33 | **0.002** |
| **meanOccurrence (/s)** | | | | | |
| A | 5.602(4.468, 6.858) | 4.520(4.051, 5.099) | 5.049(3.848, 6.258) | 19.44 | **< 0.001** |
| B | 5.212(4.483, 6.657) | 4.972(4.052, 6.066) | 4.793(3.060, 6.312) | 7.35 | **0.003** |
| C | 5.013(3.920, 6.450) | 4.098(3.573, 4.780) | 4.797(3.993, 6.569) | 14.92 | **< 0.001** |
| D | 3.439(1.488, 4.533) | 3.846(3.028, 5.454) | 3.884(2.263, 4.561) | 6.00 | **0.009** |
| F | 4.792(3.210, 6.196) | 4.417(3.921, 5.209) | 4.691(3.317, 7.427) | 1.50 | 0.239 |
| E | 5.313(4.304, 6.473) | 4.813(3.839, 5.507) | 4.390(2.741, 5.859) | 19.52 | **< 0.001** |
| G | 3.892(2.722, 5.002) | 4.025(3.765, 4.603) | 3.854(1.564, 4.532) | 2.24 | 0.130 |
| *Note: Data are expressed as median (minimum, maximum). One-way repeated measure ANOVA was used to compare differences across conscious states under ciprofol-induced anesthesia. Indicators A–G correspond to seven microstates (detailed in Methods). P < 0.05 indicates significant group differences.* | | | | | |

Table S8. Classification performance of **SVM classifiers** using frequency-specific EEG microstate features and **5-fold cross-validation**. The values are expressed as mean ± stander error.

| Features | Accuracy | Micro-F1 | F1-Wake | F1-LOC | F1-ROC |
| --- | --- | --- | --- | --- | --- |
| Broadband | 0.78±0.08 | 0.77±0.08 | 0.67±0.12 | 0.93±0.09 | 0.71±0.09 |
| Delta band | 0.64±0.12 | 0.60±0.11 | 0.73±0.19 | 0.73±0.15 | 0.33±0.24 |
| Theta band | 0.75±0.17 | 0.74±0.18 | 0.74±0.18 | 0.83±0.15 | 0.65±0.29 |
| Alpha band | 0.81±0.04 | 0.81±0.05 | 0.81±0.10 | 0.88±0.03 | 0.73±0.08 |
| Beta band | 0.63±0.12 | 0.60±0.12 | 0.66±0.12 | 0.74±0.13 | 0.40±0.27 |
| Gamma band | 0.80±0.12 | 0.79±0.12 | 0.84±0.15 | 0.87±0.11 | 0.67±0.16 |
| All sub-frequency bands | **0.96±0.04** | **0.96±0.04** | **0.98±0.05** | **0.96±0.06** | **0.93±0.07** |
| All frequency bands | 0.94±0.06 | 0.94±0.07 | 0.97±0.08 | 0.96±0.06 | 0.89±0.14 |

Table S9. Classification performance of **Random Forest classifiers** using frequency-specific EEG microstate features and **leave-one-out cross-validation**.

| Features | Accuracy | Micro-F1 | F1-Wake | F1-LOC | F1-ROC |
| --- | --- | --- | --- | --- | --- |
| Broadband | 0.768 | 0.765 | 0.681 | 0.917 | 0.698 |
| Delta band | 0.696 | 0.662 | 0.784 | 0.750 | 0.452 |
| Theta band | 0.768 | 0.762 | 0.756 | 0.846 | 0.683 |
| Alpha band | 0.826 | 0.821 | 0.880 | 0.851 | 0.732 |
| Beta band | 0.623 | 0.587 | 0.745 | 0.731 | 0.286 |
| Gamma band | 0.812 | 0.805 | 0.816 | 0.863 | 0.737 |
| All sub-frequency bands | **0.928** | **0.927** | **0.939** | **0.936** | **0.905** |
| All frequency bands | 0.913 | 0.911 | 0.898 | 0.958 | 0.878 |

Table S10. Classification performance of **Random Forest classifiers** using frequency-specific EEG microstate features and **5-fold cross-validation**. The values are expressed as mean ± stander error.

| Features | Accuracy | Micro-F1 | F1-Wake | F1-LOC | F1-ROC |
| --- | --- | --- | --- | --- | --- |
| Broadband | 0.77±0.09 | 0.76±0.10 | 0.68±0.16 | 0.92±0.08 | 0.67±0.15 |
| Delta band | 0.70±0.08 | 0.67±0.07 | 0.78±0.14 | 0.78±0.08 | 0.45±0.11 |
| Theta band | 0.72±0.14 | 0.71±0.16 | 0.65±0.19 | 0.86±0.10 | 0.62±0.25 |
| Alpha band | 0.84±0.03 | 0.84±0.03 | 0.88±0.03 | 0.87±0.04 | 0.77±0.07 |
| Beta band | 0.65±0.16 | 0.62±0.17 | 0.75±0.19 | 0.75±0.09 | 0.34±0.27 |
| Gamma band | 0.80±0.14 | 0.78±0.14 | 0.92±0.09 | 0.81±0.13 | 0.64±0.24 |
| All sub-frequency bands | **0.94±0.03** | **0.94±0.03** | **0.98±0.04** | **0.94±0.05** | **0.90±0.06** |
| All frequency bands | 0.93±0.07 | 0.92±0.08 | 0.89±0.15 | 0.96±0.06 | 0.91±0.09 |
